# Longer lifespan in male mice treated with a weakly estrogenic agonist, an antioxidant, an α‐glucosidase inhibitor or a Nrf2‐inducer

**DOI:** 10.1111/acel.12496

**Published:** 2016-06-16

**Authors:** Randy Strong, Richard A. Miller, Adam Antebi, Clinton M. Astle, Molly Bogue, Martin S. Denzel, Elizabeth Fernandez, Kevin Flurkey, Karyn L. Hamilton, Dudley W. Lamming, Martin A. Javors, João Pedro de Magalhães, Paul Anthony Martinez, Joe M. McCord, Benjamin F. Miller, Michael Müller, James F. Nelson, Juliet Ndukum, G. Ed. Rainger, Arlan Richardson, David M. Sabatini, Adam B. Salmon, James W. Simpkins, Wilma T. Steegenga, Nancy L. Nadon, David E. Harrison

**Affiliations:** ^1^ Geriatric Research, Education and Clinical Center and Research Service, South Texas Veterans Health Care System, Department of PharmacologyThe University of Texas Health Science Center at San AntonioSan AntonioTX78229USA; ^2^ Barshop Institute for Longevity and Aging StudiesThe University of Texas Health Science Center at San AntonioSan AntonioTX78229USA; ^3^ Department of Pathology and Geriatrics CenterUniversity of MichiganAnn ArborMI48109‐2200USA; ^4^ Max Planck Institute for Biology of AgeingCologneD‐50931Germany; ^5^ The Jackson LaboratoryBar HarborME04609USA; ^6^ Colorado State UniversityFort CollinsCO80523USA; ^7^ Department of MedicineUniversity of Wisconsin‐MadisonMadisonWI53705USA; ^8^ Department of PsychiatryUniversity of Texas Health Science Center at San AntonioSan AntonioTX78229USA; ^9^ School of Biological SciencesUniversity of LiverpoolCrown StreetLiverpoolL69 7ZBUK; ^10^ Division of Pulmonary Sciences and Critical Care MedicineUniversity of ColoradoAuroraCOUSA; ^11^ Norwich Medical SchoolUniversity of East AngliaNorwichUK; ^12^ Department of Physiology and Barshop Center for Longevity and Aging StudiesThe University of Texas Health Science Center at San AntonioSan AntonioTX78229USA; ^13^ Centre for Cardiovascular SciencesSchool of Clinical and Experimental MedicineThe Medical SchoolThe University of BirminghamBirminghamUK; ^14^ Department of Geriatric MedicineUniversity of Oklahoma Health Science CenterOklahoma CityOK73104USA; ^15^ VA Medical CenterOklahoma CityOK73104USA; ^16^ Whitehead Institute for Biomedical ResearchCambridgeMA02142USA; ^17^ Department of BiologyMITCambridgeMA02139USA; ^18^ Howard Hughes Medical InstituteMITCambridgeMA02139USA; ^19^ Broad Institute of Harvard and MITSeven Cambridge CenterCambridgeMA02142USA; ^20^ The David H. Koch Institute for Integrative Cancer Research at MITCambridgeMA02139USA; ^21^ Department of Molecular Medicine and Barshop Institute for Longevity and Aging StudiesThe University of Texas Health Science Center at San AntonioSan AntonioTX78229USA; ^22^ Center for Basic & Translational Stroke ResearchWest Virginia UniversityMorgantownWV26506USA; ^23^ Division of Human NutritionWageningen University and Research CentreWageningenThe Netherlands; ^24^ Division of Aging BiologyNational Institute on AgingBethesdaMD20892USA; ^25^ Present address: Integrative Genomics of Ageing GroupInstitute of Ageing and Chronic DiseaseUniversity of LiverpoolL7 8TX, LiverpoolUnited Kingdom

**Keywords:** acarbose, fish oil, metformin, NDGA, Protandim, rapamycin, UDCA, 17‐α‐estradiol

## Abstract

The National Institute on Aging Interventions Testing Program (ITP) evaluates agents hypothesized to increase healthy lifespan in genetically heterogeneous mice. Each compound is tested in parallel at three sites, and all results are published. We report the effects of lifelong treatment of mice with four agents not previously tested: Protandim, fish oil, ursodeoxycholic acid (UDCA) and metformin – the latter with and without rapamycin, and two drugs previously examined: 17‐α‐estradiol and nordihydroguaiaretic acid (NDGA), at doses greater and less than used previously. 17‐α‐estradiol at a threefold higher dose robustly extended both median and maximal lifespan, but still only in males. The male‐specific extension of median lifespan by NDGA was replicated at the original dose, and using doses threefold lower and higher. The effects of NDGA were dose dependent and male specific but without an effect on maximal lifespan. Protandim, a mixture of botanical extracts that activate Nrf2, extended median lifespan in males only. Metformin alone, at a dose of 0.1% in the diet, did not significantly extend lifespan. Metformin (0.1%) combined with rapamycin (14 ppm) robustly extended lifespan, suggestive of an added benefit, based on historical comparison with earlier studies of rapamycin given alone. The α‐glucosidase inhibitor, acarbose, at a concentration previously tested (1000 ppm), significantly increased median longevity in males and 90th percentile lifespan in both sexes, even when treatment was started at 16 months. Neither fish oil nor UDCA extended lifespan. These results underscore the reproducibility of ITP longevity studies and illustrate the importance of identifying optimal doses in lifespan studies.

## Introduction

Interventions that delay aging may provide new tools to probe the physiological processes and biochemical pathways that modulate aging and potentially lead to the development of interventions that can increase healthy lifespan in people. The National Institute on Aging Interventions Testing Program (ITP) represents a multisite translational research program to evaluate agents hypothesized to extend mouse lifespan by retarding aging and postponing late life diseases. Interventions proposed by multiple collaborating scientists from the research community are tested, in parallel, at three sites: The Jackson Laboratory (TJL), University of Michigan (UM), and the University of Texas Health Science Center at San Antonio (UT), using identical, standardized protocols in genetically heterogeneous (UM‐HET3) mice (Nadon *et al*., 2016).

Fifty‐three lifespan experiments, involving 30 test agents, have been initiated in the first 11 years of the ITP. Significant effects on longevity, in one or both sexes, have been published for 6 of the tested agents: aspirin (Strong *et al*., 2008), nordihydroguaiaretic acid (NDGA) (Strong *et al*., 2008; Harrison *et al*., 2014), rapamycin (Harrison *et al*., 2009; Miller *et al*., 2011, 2014), acarbose (ACA) (Harrison *et al*., 2014), methylene blue (Harrison *et al*., 2014), and 17‐α‐estradiol (17aE2) (Harrison *et al*., 2014). Here, we report survival analyses for mice treated with additional test agents, including ursodeoxycholic acid (UDCA), Protandim (Prot) and fish oil (FO), metformin (Met), or with the combination of Met plus rapamycin (Rapa). We also report completed lifespan analyses of mice treated with two compounds, 17aE2 and NDGA, at doses higher or lower than those tested previously and with one compound, ACA, started later in life than reported in an earlier study (Harrison *et al*., 2014).

The interventions tested in this study were selected for the following reasons:

17‐α‐estradiol (17aE2) is an optical isomer of 17‐β‐estradiol with reduced affinity for estrogen receptors. This form of estrogen is reported to be neuroprotective *in vitro* in cultured cells and *in vivo* in an ischemia–reperfusion animal model (Perez *et al*., 2005). It also has been reported to protect against neurodegeneration in cell and animal models of Parkinson's disease (Dykens *et al*., 2005) and cerebrovascular disease (Liu *et al*., 2005). We previously reported that treatment with 17aE2, at a concentration of 4.8 ppm in food, increased median lifespan in males using data pooled data from all three sites, but we noted dramatic site‐to‐site variation, with a 28% increase in median at the UT site but much smaller (3%) increases at the other two sites (Harrison *et al*., 2014). 17aE2, at 4.8 ppm, did not extend lifespan in female mice. To clarify the effects of this agent, we have now repeated the longevity study at a higher dose of 17aE2 (14.4 ppm) in both sexes.

Protandim (Prot) is a mixture of five botanical extracts, including bacosides, silymarin, withaferin A, epigallocatechin‐3‐gallate, and curcumin. This composition was designed to stimulate Nrf2/ARE activation at low concentrations of each of the compounds, in principle providing strong, synergistic Nrf2 activation with minimized off‐target side effects (Velmurugan *et al*., 2009). Previous studies have shown that in healthy humans supplemented orally with Prot over 120 days, superoxide dismutase (SOD) was increased in red blood cells by 30%, and catalase increased by 54% (Nelson *et al*., 2006). Furthermore, biochemical and histological studies in mice revealed that feeding a Prot‐supplemented diet suppressed tumor promoter‐induced oxidative stress, cell proliferation, and inflammation (Liu *et al*., 2009). Moreover, Prot treatment was reported to protect the heart from oxidative stress and fibrosis in a rodent model of pulmonary hypertension (Bogaard *et al*., 2009).

Metformin (Met) is an oral antidiabetic drug that has been FDA‐approved for the treatment of type 2 diabetes (Nathan *et al*., 2006). Treatment with Met lowers blood glucose, inhibits lipolysis, and decreases circulating free fatty acids, while producing few undesired side effects in people (Witters, 2001). Previous studies have shown that Met also inhibits the mammalian target of rapamycin (mTOR) signaling pathway, resulting in decreased phosphorylation of the mTOR complex 1 (mTORC1) substrates S6K1 and 4E‐BP1 and decreased protein translation (Dowling *et al*., 2007). Studies in both vertebrates and invertebrates have been reported showing that Met increases longevity. For example, Met was recently shown to extend both the lifespan and healthspan of the nematode *C. elegans* (Onken & Driscoll, 2010). Met has also been shown to extend the lifespan of short‐lived, tumor‐prone, HER2/neu mice and female SHR outbred mice (Anisimov *et al*., 2005). More recently, de Cabo and colleagues reported that 0.1% Met in the diet (i.e. at 1000 ppm) increased mean lifespan by 4–6% in male C57BL/6J.Nia mice (Martin‐Montalvo *et al*., 2013) and also improved indices of health. Female mice were not tested in the de Cabo study. Conversely, a study of Met on the lifespan of Fisher‐344 rats found no effect on lifespan (Smith *et al*., 2010).

Rapamycin (Rapa) and Met each inhibit mTORC1, but through distinct mechanisms. In addition, Rapa affects glucose tolerance through inhibition of a second mTOR complex, mTORC2, resulting in hepatic insulin resistance (Lamming *et al*. 2013), while Met acts to activate AMPK, suppressing hepatic glucose output (DeFronzo *et al*., 1991; Zhou *et al*., 2001). Met would be expected to diminish the negative effects of Rapa on hepatic glucose output and thus potentially lead to a survival benefit greater than that produced by Rapa alone.

Ursodeoxycholic acid (UDCA) was chosen for testing because bile acids (BAs), endogenous products of cholesterol catabolism, have been reported to be associated with increased xenobiotic detoxification gene expression in long‐lived rodents (e.g. Amador‐Noguez *et al*., 2007). Moreover, in a clinical trial UDCA prevented colorectal adenoma recurrence (Alberts *et al*., 2005). UDCA treatment suppressed chemically induced diabetes in rats (Lukivskaya *et al*., 2004). Thus, UDCA has a number of properties that suggested it might have anti‐aging effects, including increased xenobiotic stress resistance, protection from metabolic derangements such as diabetes, and suppression of tumor formation.

Nordihydroguaiaretic (NDGA) is produced by the leaves of the creosote bush (*Larrea tridentata*). It is both a lipoxygenase inhibitor and potent antioxidant. In earlier reports, it was shown to increase median lifespan, but only in male mice (Strong *et al*., 2008; Harrison *et al*., 2014). The sex difference was tentatively attributed to differences in pharmacokinetics, as male mice maintained higher trough blood levels of NDGA than females. To test this interpretation, and to define the optimal NDGA concentration for lifespan extension in males, we repeated the study starting at 6 months of age at the dose used in the original study, 2500 ppm, plus doses higher (5000 ppm) and lower (800 ppm) than tested previously. An interim survival analysis was published (Harrison *et al*., 2014), reporting the effects of the three doses on median lifespans in male mice and the highest dose on median lifespan in female mice. Here, we report the completed survival analyses, including the effects on maximal lifespan.

Fish oil (FO) was selected for testing, at two doses, in part because several epidemiologic studies have associated reduced risk of cardiovascular disease with fish or fish oil consumption (e.g. Kris‐Etherton *et al*., 2002). FO has also been shown to reduce several risk factors associated with coronary heart disease (Sidhu, 2003) such as lowering triglyceride levels (Kris‐Etherton *et al*., 2002). Additionally, studies suggest that consumption of FO or of its omega‐3 fatty acids may have beneficial effects on stroke, depression, cancer, and Alzheimer's disease (Rose & Connolly, 1999; Sidhu, 2003).

Acarbose (ACA) is an inhibitor of intestinal α‐glucosidase. It inhibits digestion of polysaccharides and attenuates the uptake of sugars from the GI tract. It lowers postprandial glucose excursions and is prescribed for the treatment of type 2 diabetes. It was selected for testing in part because it was proposed to act as a dietary restriction mimetic. In our earlier report, ACA increased median and maximal lifespan in both male and female UM‐HET3 mice (Harrison *et al*., 2014). In that study, treatment was begun at 4 months of age. Because it is more clinically relevant to begin anti‐aging treatments late in life, we initiated a study in which ACA treatment began at 16 months of age.

## Results

### 17‐α‐estradiol (17aE2)

**Figure 1:**
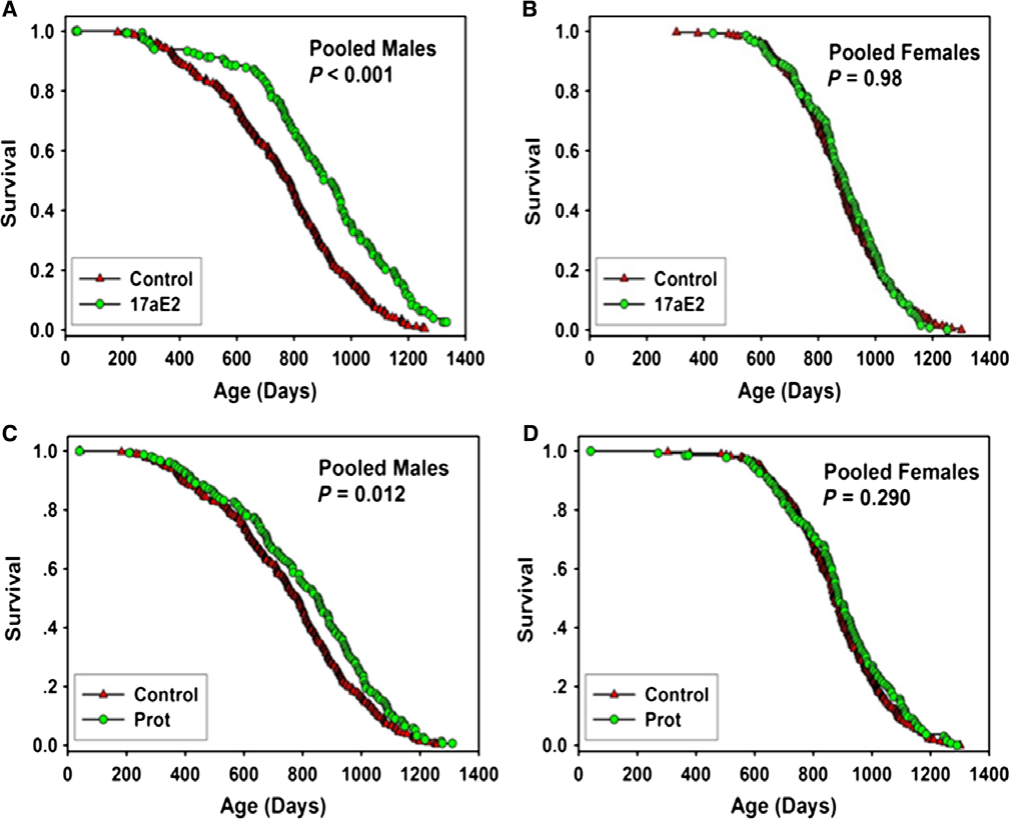
Survival curves for mice treated with 17‐α‐estradiol (17aE2) or Protandim (Prot) pooled across sites. (A) males treated with 14 ppm 17aE2. (B) females treated with 14 ppm 17aE2. (C) males treated with Prot at 600 ppm (10–17 months of age) followed by 1200 ppm (17 months of age on). (D) females treated with Prot at 600 ppm (10–17 months of age) followed by 1200 ppm (17 months of age on). Each symbol represents one mouse. *P*‐values reflect log‐rank test, stratified by test site. See Table 1 for statistical results.

Beginning at 10 months of age, male and female mice were fed chow containing 17aE2 at a concentration of 14.4 ppm (17aE2). As shown in Table 1 and Figure 1, median lifespan increased 19% in male mice, calculated from data pooled from the three sites (*P* < 0.001). The effects of 17aE2 were significant at all three sites, at *P* < 0.003, with increases in median survival of 26%, 9%, and 23% at TJL, UM, and UT (Fig. S1, Supporting information). The age at which 90% of the mice had died increased on average by 12% (21%, 8%, and 8% at the three sites; Table S1, Supporting information), and the difference in survival at the 90th percentile was significantly increased (*P* < 0.001 for the pooled data; Table 1). 17aE2 treatment had no significant effect on female survival, either in the pooled data or at any individual site (Table 1, Table S1, Supporting information, Fig. 1 and Fig. S1, Supporting information). Interestingly, survival of male 17aE2‐treated mice was also significantly higher than survival for the female control group (*P* < 0.0006) and for the female 17aE2‐treated group (*P* < 0.0135).

**Table 1:** C2011 mice, pooled across sites

Group	Number	Median days	Median % increase	Log‐rank *P*‐value	90th percentile days	90th percentile increase	Wang–Allison *P*‐value
Males: pooled across sites							
Control	294	780			1064		
17aE2	144	925	19	0.000	1193	12	0.000
Prot	155	834	7	0.012	1130	6	0.103
Met	148	835	7	0.348	1046	−2	0.405
Met/Rapa	158	959	23	0.000	1175	10	0.000
UDCA	149	832	7	0.448	1063	0	0.868
Females: pooled across sites							
Control	281	874			1092		
17aE2	135	883	1	0.981	1091	0	0.864
Prot	134	896	3	0.291	1154	6	0.163
Met	140	872	0	0.791	1094	0	0.617
Met/Rapa	142	1078	23	0.000	1278	17	0.000
UDCA	133	865	−1	0.762	1105	1	0.491

Although 17aE2 is generally thought to be ‘non‐feminizing’, there is evidence that 17aE2 can have uterotrophic effects (Clark *et al*., 1982). 17aE2 at 4.8 ppm, as used in our previous report (Harrison *et al*., 2014), had no significant effects on uterine weight when fed to ovariectomized mice (Fig. S2, Supporting information, *P* = 0.44). However, we considered the possibility that it might be uterotrophic at the higher dose (14 ppm) used in our current study. We therefore tested for estrogenic effects of the 17aE2 at 14 ppm in young‐ and middle‐aged ovariectomized UM‐HET3 mice bred at UT. As shown in Figure S2 (Supporting information), the 14.4 ppm 17aE2 diet fed to ovariectomized mice for 6 weeks increased uterine weight to a level statistically indistinguishable from that of intact controls. Additionally, mice fed the high dose had mean uterine weights significantly higher than that of the ovariectomized control group (*P* = 0.0014).

### Protandim (Prot)

**Figure 2:**
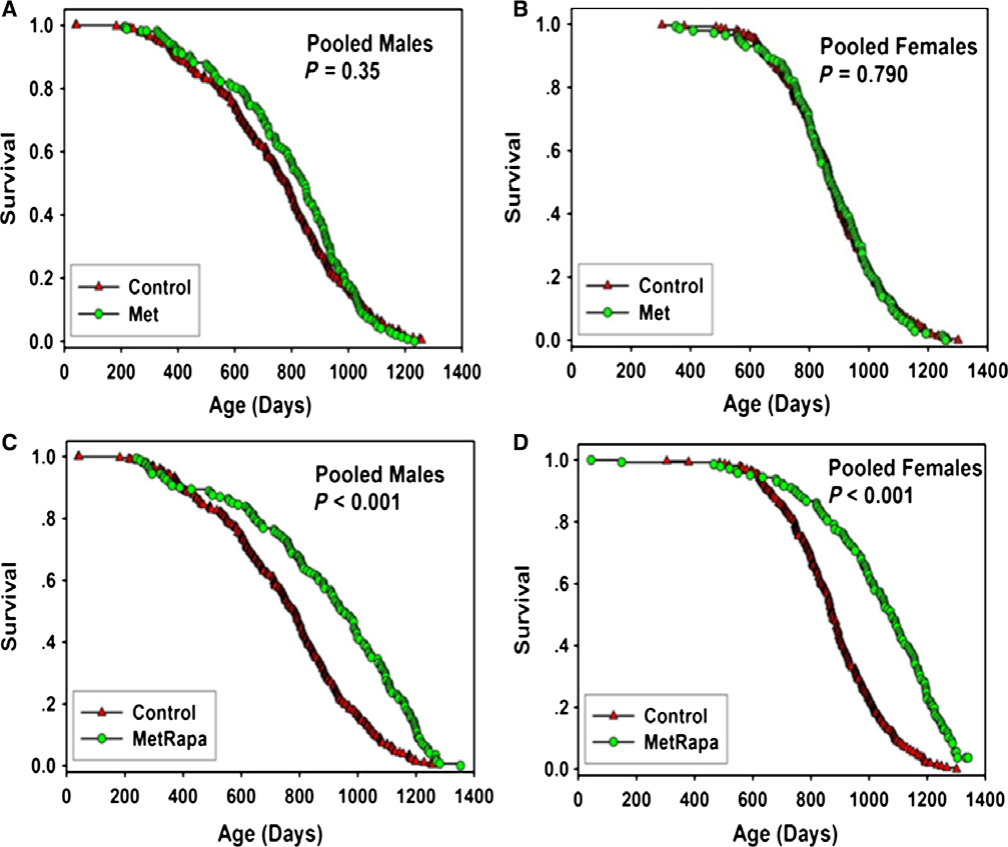
Survival curves for mice treated with metformin (Met) with and without rapamycin (Rapa), pooled across sites. (A) males treated with 1000 ppm Met. (B) females treated with 1000 ppm Met. (C) males treated with 1000 ppm Met plus 14 ppm Rapa. (D) females treated with 1000 ppm Met plus 14 ppm Rapa. Each symbol represents one mouse. *P*‐values reflect log‐rank test, stratified by test site. See Table 1 for statistical results.

Beginning at 10 months of age, one group of mice was fed chow containing Prot at 600 ppm, to approximate the intake of humans who consume this commercially available nutritional supplement. The dose of Prot was increased from 600 to 1200 ppm when the mice reached 17 months of age, because testing of mice not included in the longevity cohort had indicated that 600 ppm, contrary to expectation, did not modulate levels of liver mRNA for genes involved in xenobiotic responses. As shown in Figure 1 and Table 1, there was a significant effect (*P* < 0.012) of Prot on survival of male mice in the pooled population, with a 7% increase in median survival. There was no significant difference in the proportion of control and Prot mice alive at the age at 90% mortality (*P* = 0.1 by the ‘Wang–Allison test’). A secondary analysis of the survival data from individual sites revealed a significant increase in median survival (*P* = 0.03) at the UT site but no significant effects at the TJL and UM sites (Fig. S3 and Table S1, Supporting information). There were no significant effects of Prot on median or maximal survival of females in the pooled data (Table 1, Fig. 2) or in the data from any individual site (Table S1, Fig. S3, Supporting information).

### Metformin alone or with rapamycin (Met or Met/Rapa)

**Figure 3:**
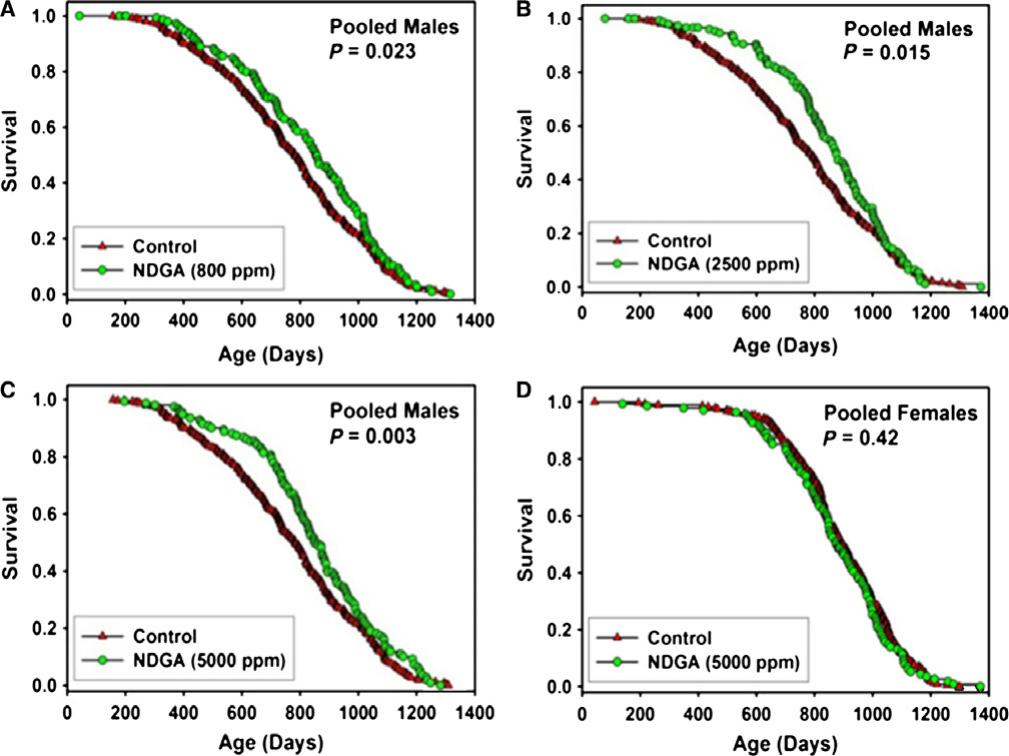
Survival curves for mice treated with NDGA, pooled across sites. (A) males treated with 800 ppm. (B) males treated with 2500 ppm. (C) males treated with 5000 ppm. (D) females treated with 5000 ppm. Each symbol represents one mouse. *P*‐values reflect log‐rank test, stratified by test site. See Table 2 for statistical results.

Beginning at 9 months of age, groups of male and female mice began consuming a diet containing 1000 ppm (0.1%) Met. As shown in Figure 2 and Table 1, Met led to a 7% increase in median lifespan of males when data were pooled across sites, but the effect was not statistically significant (*P* = 0.35). Males treated with Met had site‐specific changes of 13%, −1%, and 10% at the three test sites, but none of these was statistically significant (Table S1, Supporting information; see Fig. S4, Supporting information for the site‐specific survival curves). There was no effect of Met on survival of female mice, either in the pooled data (Table 1, Fig. 3), or at any site (Table S1, Fig. S4, Supporting information).

Figure 2 shows survival curves of male and female mice that received both Met (1000 ppm) and Rapa (14 ppm) (Met/Rapa) from 9 months of age. As shown in Table 1, this combination led to a 23% increase in survival compared with controls in both sexes, as well as to significant increases in maximum lifespan (*P* < 0.001). Met/Rapa led to a significant increase in lifespan in both sexes at each site separately (Table S1 and Fig. S5, Supporting information) and to significant increases in maximal lifespan except for UT males.

Although the current (C2011) cohort did not contain any mice given Rapa alone, we thought it would be of interest to compare the survival of mice receiving both Rapa and Met to survival of mice treated with the same Rapa dose in previous years, C2006 and C2009 (Miller *et al*., 2011, 2014). Results are shown in Table S4 (Supporting information). Males given Met/Rapa had a 23% increase in median longevity, higher than the 10% effect produced by Rapa alone in C2006 or the 13% effect in C2009 males. When the results from C2006 and C2009 males were combined for optimal statistical power, and compared with survival in C2011 mice receiving Met/Rapa, the difference did not reach statistical significance (*P* = 0.12) using our standard calculation, which stratifies by site. An alternate analysis, omitting site stratification, found *P* = 0.049 for the contrast between Met/Rapa and the historical datasets using Rapa alone at the same dose. Female mice given Met/Rapa also had a higher percentage increase in median lifespan (23%) than females that had received rapamycin alone in the previous C2006 and C2009 cohorts (18% and 21%, respectively), but the difference did not reach significance by log‐rank testing.

### Nordihydroguaiaretic acid (NDGA)

In a previous study, we tested the effects of NDGA on survival of male and female UM‐HET3 mice at a dose of 2500 ppm and reported that median lifespan was increased in males by 12%, with no benefit in female mice (Strong *et al*., 2008). In the earlier study, 9% of male control mice were alive at the joint 90th percentile age, compared with 13% of NDGA‐treated males (*P* = 0.12). Female mice given 2500 ppm had lower blood levels of NDGA compared with males, suggesting that the sex effect might reflect sex‐specific differences in drug metabolism or excretion (Strong *et al*., 2008). It was also unclear whether the optimal NDGA dose in males might be higher or lower than 2500 ppm. In the current study, therefore, we tested the effects of NDGA at 800, 2500, and 5000 ppm in males and at 5000 ppm in females, starting treatment at 6 months. Our previous (Harrison *et al*., 2014) report showed that median lifespan was increased at each dose in males and that there was no effect of the high dose on median lifespan in females, despite the fact that the highest dose in females produced a blood level equivalent to that of males fed the original 2500 ppm dose. Here, we report the completed lifespan analyses. As shown in Figure 3 and Table 2, NDGA increased survival of male mice at each of the doses tested, with increases in median lifespan at each dose (as reported previously in Harrison *et al*., 2014). There was, however, no effect of NDGA treatment on maximal lifespan at any dose tested in males, or in females at the 5000 ppm dose tested (Table 2). There was also no effect on maximal lifespan at any of the individual sites at any dose (Table S2, Supporting information).

**Table 2:** C2010 and C2012 mice, pooled across sites

Group	Number	Median days	Median % increase	Log‐rank *P*‐value	90th percentile days	90th percentile increase	Wang–Allison *P*‐value
C2010							
Males: pooled across sites							
Control	274	780			1085		
FO (50 000)	146	749	−2	0.218	1040	−4	0.317
FO (15 000)	141	830	7	0.263	1078	0	0.864
NDGA (5000)	137	839	9	0.003	1108	2	0.087
NDGA (2500	133	851	10	0.015	1080	0	0.864
NDGA (800)	132	831	6	0.023	1103	2	0.864
Females: pooled across sites							
Control	264	900			1111		
FO (50 000)	135	919	2	0.246	1084	−2	0.481
FO (15 000)	132	852	−5	0.090	1084	−2	0.481
NDGA (5000)	125	874	−3	0.416	1077	−3	0.594
C2012							
Males: pooled across sites							
Control	283	823			1055		
ACA	147	875	6	0.000	1183	12	0.0001
Females: pooled across sites							
Control	278	881			1100		
ACA	135	902	2	0.07	1166	6	0.010

**Figure 4:**
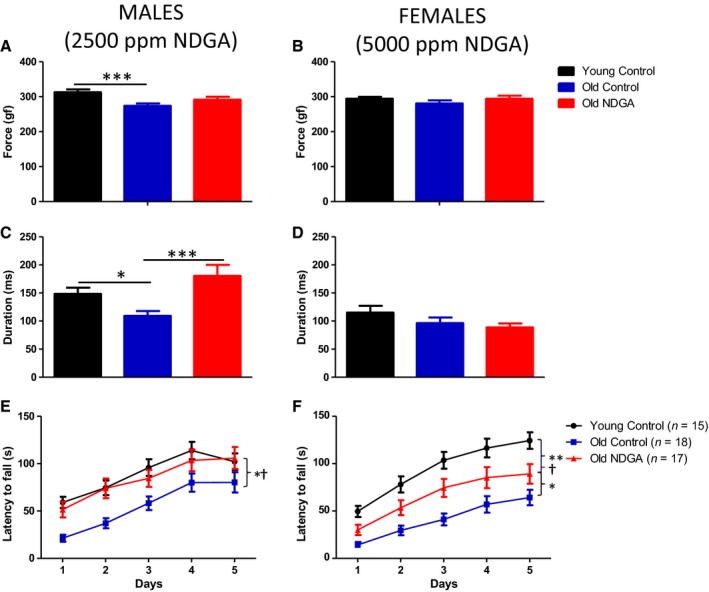
Effects of NDGA on grip strength and duration and on rotarod performance. Panels A,C,E: males. Panels B,D,F: females. The data are expressed as mean ± SEM of the number of mice shown in parentheses. *significantly different from old NDGA; ^†^significantly different from young controls; ***significantly different from old control.

We have also now assessed the effects of NDGA on neuromuscular performance. Male mice were given 2500 ppm and females given 5000 ppm from 13 months of age, and then tested at 22 months of age, together with age‐matched controls and young (4 month old) control mice. Aging impaired grip strength and grip duration in old male control mice (*P* < 0.05) as compared to young males. Grip duration of male NDGA mice was indistinguishable from that of young mice and significantly higher than that of old control males (Fig. 4). In the rotarod tests, using a mixed‐effects model that compensated for day of testing, latency to fall in young mice, as expected, was higher than in aged control mice in each sex (*P* = 0.01 for males, *P* < 0.001 for females). Fall latency was increased significantly by NDGA in both male and female mice at age 22 months (Fig. 4; *P* = 0.02 in each sex).

### Fish oil (FO)

**Figure 5:**
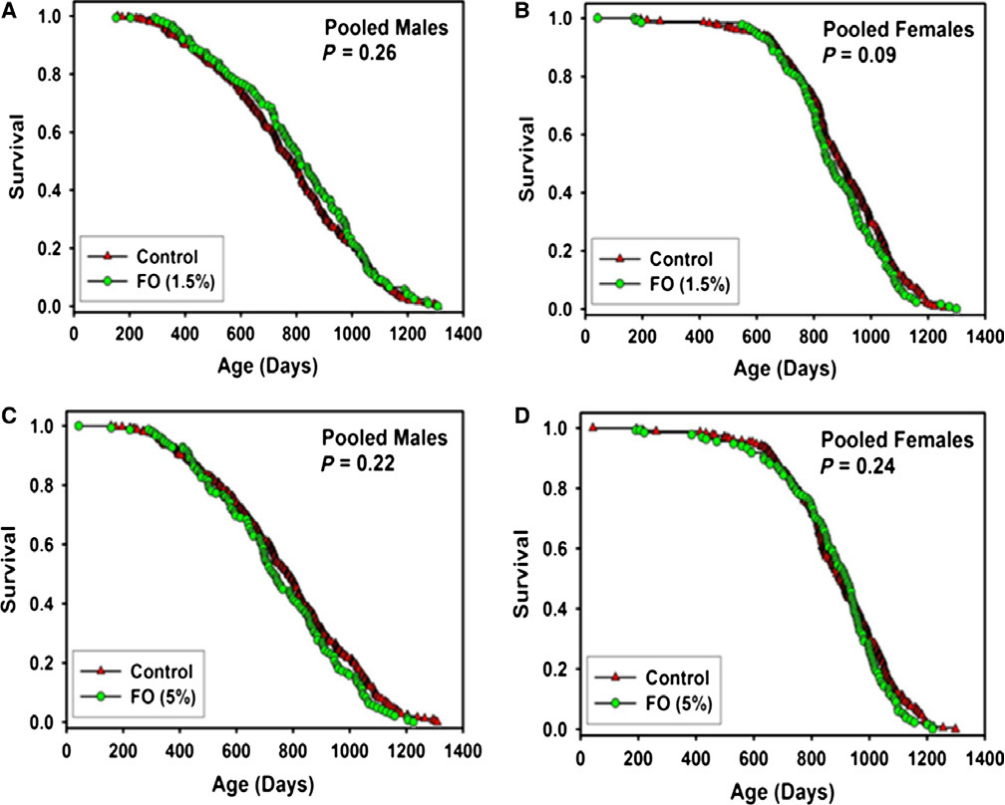
Survival curves for mice treated with fish oil (FO), pooled across sites. (A) males treated with 15 000 ppm (B) females treated with 15 000 ppm. (C) males treated with 50 000 ppm. (D) females treated with 50 000 ppm. Each symbol represents one mouse. *P*‐values reflect log‐rank test, stratified by test site. See Table 2 for statistical results.

Results of FO, at 15 000 or 50 000 ppm from 9 months of age, are shown in Figure 5 and Table 2. Pooling across sites, there were no significant effects at either dose of FO on survival of male or female mice. Additionally, there were no significant benefits of FO in either sex at any individual site (Table S2, Supporting information). Fish oil treatment was associated with a significant (*P* < 0.05) dose‐dependent increase in body weight in males at 18 months of age, but no significant effects on body weight in females (Fig. S8, Supporting information). It is notable that FO, at the higher dose, led to a significant decline in male longevity at UM (−18%, *P* = 0.003, and that the lower FO dose led to a 9% increase in male lifespan (*P* = 0.06) at UT (Table S2, Supporting information), helping to emphasize the value of simultaneous testing with parallel protocols at all three ITP sites and the perils of relying on longevity tests conducted with a single dose at a single site.

### Ursodeoxycholic acid (UDCA)

**Figure 6:**
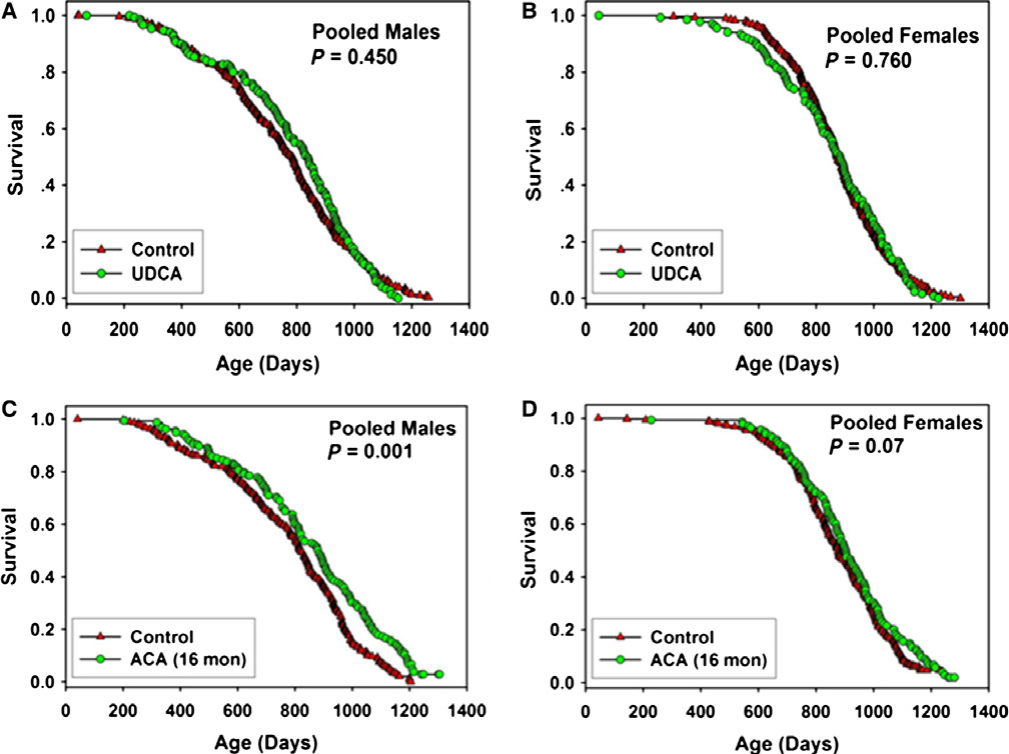
Survival curves for mice treated with ursodeoxycholic acid (UDCA)or acarbose (ACA), pooled across sites. (A) males treated with 5000 ppm UDCA. (B) females treated with 5000 ppm UDCA. (C) males treated with 1000 ppm ACA. (D) females treated with 1000 ppm ACA. Each symbol represents one mouse. *P*‐values reflect log‐rank test, stratified by test site. See Tables 1 and 2 for statistical results.

Beginning at 5 months of age, mice were fed chow containing 5000 ppm UDCA. The results are shown in Figure 6 and Table 1, with site‐specific statistics in Table S1 (Supporting information). Pooling the data across sites, there were no significant differences in survival for male or female mice given UDCA, and no significant benefits at any site considered separately (Table S1, Supporting information). On the other hand, pooling across sites there were significant effects of UDCA on body weights in males and female mice as shown in Figure S7 (Supporting information). Body weighs of UDCA‐fed mice were significantly (*P* < 0.001) lower than control at 12 and 18 months in male mice and at 12, 18, and 24 months of age in female mice.

### Acarbose (ACA)

Beginning at 16 months of age, mice were fed chow containing 1000 ppm ACA. The results are shown in Figure 6 and Table 2 with site‐specific statistics in Table S3 (Supporting information). Pooling the data across sites, there was a significant increase in survival for male mice treated with ACA, with a 6% increase in median lifespan and a significant 12% increase in maximal lifespan (see Fig. 6 and Table 2). ACA started at this late age had only a small effect on median lifespan in females (2%, *P* = 0.07), but led to a significant (6%, *P* = 0.01) increase in maximal lifespan.

## Discussion

This paper provides new and updated longevity results for eight compounds and mixtures tested by the ITP. 17aE2, previously shown to provide modest and variable improvements in male lifespan, has now been tested at a higher dose, and shown to produce dramatic and consistent improvement in male longevity, but no benefits to female mice. 17aE2 males live 19% longer than control males and longer than control or 17aE2‐treated females. Prot, a mixture that activates Nrf2, led to a small but statistically significant increase (7%) in males, without a significant improvement in survival to the 90th percentile age. On its own, Met did not produce significant lifespan benefits in either sex. The combination of Met/Rapa increased lifespan beyond the levels seen in previous cohorts of mice given Rapa alone, particularly in male mice, but statistical significance of this historical comparison was equivocal. An interim analysis had revealed that NDGA increased lifespan of male mice significantly at each of three tested doses (Harrison *et al*., 2014) but did not increase female lifespan. The completed lifespan analysis reported here revealed that NDGA did not significantly augment maximal longevity in either sex. Male mice treated with NDGA were protected against the age‐related loss in grip duration, and mice of both sexes were protected against age‐related decline in rotarod performance. ACA, previously shown to increase median and maximal lifespan in both male and female mice when started at 4 months of age (Harrison *et al*., 2014), was tested again, with treatment started at 16 months of age, and shown to increase median lifespan in males, and maximal lifespan in both sexes. Fish oil, at either of two tested concentrations, and the bile acid UDCA produced no lifespan benefits at the tested concentrations.

In our previous study, 17aE2 at 4.8 ppm produced a significant increase in male longevity in the pooled dataset, that is, by the planned primary analysis, but a secondary analysis showed much stronger effects at UT than at the other two sites, complicating interpretation. At the higher dose (14 ppm) used in the current study, however, 17aE2 had significant effects on male mice at each site, ranging from a 9% increase at UM (*P* = 0.003) to 26% at TJL, with an average increase of 19%, only slightly less than the 23% increase produced by the highest dose of rapamycin tested in our previous work (Miller *et al*., 2014). There were no benefits seen in females, either at the lower dose previously used or at the higher dose used in the current cohort.

The mechanism underlying the sex‐specific benefit of 17aE2 is unknown. Interestingly, survival of male mice given 17aE2 was significantly higher than survival in females receiving this drug or in untreated females, suggesting that the beneficial effects in males were not simply due to recapitulation of estrogenic effects produced physiologically in normal females, in which estrogen levels vary throughout the reproductive period and show age‐related changes in the postreproductive period. The dose of 17aE2 that we used in our previous report (Harrison *et al*., 2014) had no significant effects on uterine weight when fed to ovariectomized mice (see Fig. S2, Supporting information), but the higher dose, 14 ppm, did produce significant effects on uterine weights in ovariectomized mice. This indicates that the dose of 17aE2 used in this study has bioactivity in females and therefore that the lack of an effect on female survival was not due to a lack of bioavailability in 17aE2‐fed female mice. The uterotrophic effect of this dose of 17aE2 also indicates that it acts on classical estrogen receptors, at least at higher concentrations, and could therefore modulate reproductive and other estrogen receptor‐mediated functions in males. It would be of interest to learn whether 17aE2 at either dose modulates pathways thought to be relevant to aging and lifespan, such as those linked to ATF4, mTOR, autophagy, adipokine production, inflammation, proteasome function, and others, and does so in a sex‐specific way.

It is not known whether higher doses of 17aE2 might lead to a greater degree of lifespan extension than the 19% increase documented at 14 ppm in this study. Studies of the effects of 17aE2 in ovariectomized females and castrated males, or in mice lacking the classical estrogen receptor, or in testosterone‐treated females, could also be informative. Additional data on steroid metabolism and steroid‐sensitive feedback circuits, in male and female mice, might help to clarify the basis for the effects of this agent on male mice, and provide clues as to how to achieve similar success in females. It is notable that most deaths in UM‐HET3 mice are attributable to some form of neoplasia (Miller & Chrisp, 2002; Lipman *et al*., 2004; Harrison *et al*., 2014), suggesting that studies of 17aE2 on oncogenesis and on host antitumor defenses also deserve experimental attention.

The finding that the Nrf2 activator, Protandim (Prot), increased longevity in genetically heterogeneous mice is consistent with previous reports, suggesting that Nrf2 activation may be causally associated with increased lifespan. Although we cannot rule out effects at other targets by the individual constituents of the mixture of compounds contained in Prot, the increase in expression of xenobiotic response genes in the livers of mice in the present study at the 1200 ppm dose (data not shown) further supports the idea that the effects of Prot on longevity may be at least partly due to Nrf2 activation. For example, Leiser & Miller (2010) reported that primary skin‐derived fibroblasts from the long‐lived Snell dwarf mutant mouse manifest elevated levels of Nrf2, higher levels of glutathione, and increased resistance to plasma membrane lipid peroxidation. Consistent with those findings, mRNA levels for Nrf2‐sensitive genes were reported to be elevated in selected tissues of Snell dwarf mice (Leiser & Miller, 2010). Taken together, these results suggest that Nrf2 activation may contribute to the stress resistance and increased longevity observed in the Snell dwarf mouse. In the present study, only male mice showed positive effects of Prot on longevity. This sex‐specific effect may be due to differences in how males and females metabolize and/or eliminate any or all of 5 constituents present in the mixture of compounds contained in Prot. Alternatively, there may be sex differences in the mechanisms that control the Keap1‐Nrf2 pathway. For example, Sykiotis & Bohmann (2008) reported that *D*. *melanogaster* carrying a loss of function mutation in Keap1 had increased Nrf2 activity, increased oxidative stress resistance, and elevated longevity, but in male flies only. Although male mice in the present study had a significant extension of median lifespan, they did not show an increase in maximal lifespan, suggesting that the effects of Prot are most beneficial earlier in life. Phase II studies examining the effects of doses higher and lower than those used in the present study may be informative in this regard. In addition, studies examining the response to Prot over the lifespan may also help elucidate mechanisms underlying the different effects of Prot earlier and later in the lifespan.

Metformin (Met) has been reported to increase longevity in male mice of the C57BL/6 inbred stock (Martin‐Montalvo *et al*., 2013) at a dose of 1000 ppm, started at 54 weeks of age, leading to an increase in mean lifespan of 5.8%. This increase was deemed to be significant using the Gehan–Breslow statistic, which gives greater emphasis to deaths at earlier ages than the log‐rank test used in our own work. It is not clear whether the use of the log‐rank statistic for the C57BL/6 mice would have supported the inference of a significant effect of Met. A parallel study of male B6C3F1 mice also suggested a benefit of Met, with a 4% extension of mean lifespan (Gehan–Breslow *P* = 0.06). The de Cabo group did not report any data on female mice treated with Met. Our current study used the same dose of Met (1000 ppm = 0.1%), but differs in several respects: use of genetically heterogeneous mice, initiation of Met treatment at 9 months (rather than 12 months) of age, evaluation of male and female mice, analysis at three independent sites, and use of the log‐rank statistic. Statistical power also differed: The de Cabo group used 64 Met mice and 83 control mice, while the ITP protocol used 148 Met mice and 294 controls, distributed among the three test sites. The pooled ITP data showed a non‐significant effect of Met on median lifespan (7% increase), and the site‐specific effects (13%, −1%, and 10% at TJL, UM, and UT) were also indistinguishable from chance. In the ITP dataset, the age at 90th percentile mortality declined by 2% in the Met group. In C57BL/6 mice, Met was toxic at a higher concentration of 10 000 ppm (1%) (Martin‐Montalvo *et al*., 2013), and it is possible that evaluation of doses higher or lower than the 1000 ppm dose we used might have produced stronger evidence of benefit. Observational data suggest that patients with diabetes who take metformin have lower mortality risks than age‐matched non‐diabetic patients (Bannister *et al*., 2014). These suggestive reports, together with the strong evidence that Met can be administered with few side effects over many years in people, have prompted suggestions that this agent should be used in large‐scale clinical trials to prevent age‐associated disease in non‐diabetic subjects (Hall, 2015).

The decision to test the combination of Met and Rapa was based on the idea that the disturbances in glucose homeostasis induced by Rapa might limit its benefits on lifespan (Lamming *et al*., 2013) and that Met, by increasing insulin sensitivity, might therefore compensate for potentially harmful side effects. Our new data are consistent with this idea, in that male and female mice treated with Met (at 1000 ppm) and Rapa (at 14 ppm) showed a greater percentage increase in median lifespan than in either of two previous cohorts of mice treated with Rapa (at 14 ppm) alone. Our evidence is inconclusive, however, in that the comparisons are to historical rather than to simultaneous cohorts, the effect seen in males is not statistically significant by our standard site‐stratified log‐rank test (*P* = 0.12), and the effect in females is small.

It is possible that alternate dosing regimens, including protocols that begin one or both drugs at later ages, or use other doses, or which alternate drug‐free with drug‐treatment periods, could produce more conclusive evidence of synergy between these two agents or others in their class.

The use of rapamycin analogs with a reduced impact on glucose metabolism, such as everolimus, might also be considered (Arriola Apelo *et al*., 2016). Other compounds that regulate glucose homeostasis, including the PPARγ activator rosiglitazone, might also synergize with rapamycin; rosiglitazone was recently shown to normalize fasting hyperglycemia and attenuate rapamycin‐induced glucose intolerance and insulin resistance in rats (Festuccia *et al*., 2014). Acarbose, an agent that extends both male and female lifespan and which is thought to work by preventing post‐prandial surges in blood glucose concentration (Harrison *et al*., 2014), may also deserve consideration in combination with rapamycin treatment.

Our new data on NDGA make two points of interest. First, the failure of NDGA, at 2500 ppm, to increase longevity in female mice (Strong *et al*., 2008) seems not to be due merely to lower blood concentrations of this agent in females, because we fail to see increased female lifespan even at a concentration, 5000 ppm, that produces blood levels similar to those seen in males at 2500 ppm (Harrison *et al*., 2014). In addition, our new data replicate the earlier finding of longevity increase using 2500 ppm in males and show that benefits are also seen at twofold higher and threefold lower doses. NDGA did not, however, significantly alter the proportion of mice alive at the 90th percentile at any dose, suggesting that its positive effects may be limited to the middle portion of the lifespan. The percentage increase in median male lifespan increases with dose at TJL (7%, 16%, 19%) and at UT (5%, 11%, 15%), but not at UM (7%, 2%, −7%), which may reflect the consistent, but unexplained, pattern of higher longevity in UM control males compared with control males at the other two test sites. The finding of increased median lifespan, but no increase in maximum lifespan in NDGA‐treated male B6C3F1 hybrid mice, was also recently reported by Spindler *et al*. (2015); females were not tested in the Spindler study. In the Spindler study, significant results were seen at 3500 ppm but not at 1500, 2500, or 4500 ppm. As discussed earlier for Prot, studies examining the response to NDGA over the lifespan may also help elucidate mechanisms underlying the different effects of NDGA earlier and later in the lifespan.

In contrast to the sex differences observed in NDGA's effects on lifespan, it is noteworthy that NDGA attenuated age‐related deficits in rotarod performance in both males and females. The lack of effect on NDGA on female lifespan and its positive effects on female rotarod performance supports the idea that mechanisms underlying the effects of specific interventions on lifespan may be distinct from those contributing to some age‐sensitive physiological traits. Studying a wider range of age‐sensitive traits may be informative. The activity of NDGA as an anti‐inflammatory agent with antioxidative effects opens new avenues for mechanistic testing and a tool for studying differences between male and female mice for control of aging and late‐life diseases.

Although no previous studies of the effects of FO on lifespan of long‐lived strains of mice have been reported, several studies have been reported in which omega‐3 fatty acids increased lifespan in short‐lived autoimmune‐prone (NZB × NZW)F1 mice (Jolly *et al*., 2001; Fernandes, 2008). A demographic analysis of the survival of fish oil‐fed (NZBxNZW)F(1) mice by de Magalhaes *et al*. (2005) revealed that fish oil altered the Gompertz slope, increasing the mortality rate doubling time twofold (de Magalhaes *et al*., 2005), suggesting that FO may slow aging. However, in the present study there was no effect of encapsulated FO on lifespan at either of two doses, 15 000 or 50 000 ppm, on either median or maximal lifespan. There was, however, a significant effect on body weight, at least in males, suggesting that FO was biologically active. Thus, FO may increase lifespan, but only in a disease‐specific context.

Treatment with the bile acid, UDCA, has been reported to have a wide variety of effects that are compatible with extended lifespan in mammals, including protecting against metabolic derangements such as diabetes, and suppression of tumor formation (Oyama *et al*., 2002; Lukivskaya *et al*., 2004). Nevertheless, no previous lifespan studies have been reported for UDCA. At the dose used in this study, there were significant reductions in body weight across the lifespan in both male and female mice, suggesting that UDCA treatment had biological or physiological effects. However, there was no effect of UDCA on median or maximal lifespan in either sex. Given the wide range of positive effects reported for UDCA on measures of healthspan, it is unclear why it had no effect on lifespan. It is possible that higher or lower doses of UDCA may be effective. On the other hand, as we observed with NDGA in the present study, the effects of specific interventions on lifespan may be distinct from effects on age‐sensitive physiological changes in specific organ systems.

We previously reported that ACA increased median and maximal lifespan in both male and female mice when started at 4 months of age (Harrison *et al*., 2014). We tested ACA again at the dose used previously, but with treatment started at 16 months of age. We found that starting ACA treatment later in life led to increases in median lifespan in males, and in maximal lifespan in both sexes. In the previous study, the increase in median survival calculated from the pooled data from male mice was 22%, but was only 6% in the present study. Similarly, in females, there was a significant increase in survival in the previous study, with a 5% increase in median lifespan, but no significant effect (*P* = 0.07) on lifespan in the present study. Thus, the effects of ACA treatment appear to be optimal when treatment is initiated earlier in life. We are currently testing different doses, with treatment initiated at an intermediate time (12 months of age). We are also performing cross‐sectional studies, testing the effects of ACA on age‐sensitive changes in multiple tissues. Studies like these may help us to better understand the mechanism(s) of action of ACA on lifespan and healthspan. Studies such as these may also help inform the design of human trials of ACA, which is FDA‐approved for treatment of type 2 diabetes.

The NIA ITP has matured into a useful test bed for initial investigation of drugs and mixtures that deserve evaluation for lifespan extension in mice (Nadon *et al*., 2016). The features of the ITP design, including parallel studies in males and females at each of three laboratory sites, the use of genetically heterogeneous mice to guard against conclusions based on a single, typically inbred, genotype, and the use of enough numbers of mice to provide decent statistical power for modest (e.g. 10%) lifespan benefits have now stimulated new ideas and impetus on several biogerontological fronts. ITP studies of rapamycin have helped to motivate new work on mTOR and aging in cells, mice, and even human studies, while newer observations on acarbose and, now, 17aE2 are expected to focus experimental attention on glucose transients and sex steroids as potential mediators of age‐related diseases and as potential guides to human preventive trials. Many of the drugs are known to be safe in humans (for short‐term or long‐term use), and clinical studies are easy to imagine and in some cases, for example, rapamycin and acarbose, are already underway even prior to a definitive molecular understanding of target(s). We note that some of the most impressive ITP longevity results, such as those seen with acarbose and 17aE2, reflect physiological pathways that are not easy to model in worms or flies, whose pathways of starch digestion and steroid sensitivity differ dramatically from those used by mammals. The growing availability of tissue banks from mice treated with these agents should serve as a spur toward increased collaboration on the pathways by which these drugs lead to preserved health and postponed mortality in mice.

## Experimental procedures

### Animals

UM‐HET3 mice were produced at each of the three test sites as previously described in detail (Miller *et al*., 2011; Strong *et al*., 2013; Harrison *et al*., 2014). The mothers of the test mice were CByB6F1/J, JAX stock #100009, whose female parents are BALB/cByJ and whose male parents are C57BL/6J. The fathers of the test mice were C3D2F1/J, JAX stock #100004, whose mothers are C3H/HeJ, and whose fathers are DBA/2J. For breeding cages, each site used Purina 5008 mouse chow. For weanlings prior to 4 months of age, each site used Purina 5LG6.

Mice were housed as previously described (e.g., Strong *et al*., 2013) in plastic cages with metal tops, using ¼ inch corn‐cob bedding (Bed O'Cobs, produced by The Andersons, PO Box 114, Maumee, Ohio). Mice were given free access to water, acidified (pH 2.5–2.7) by addition of hydrochloric acid, using water bottles rather than an automated watering system. Mice were housed in ventilated cages and were transferred to fresh cages every 14 days. Temperature was maintained within the range of 21–23 °C.

At the age of 42 days, each cage was assigned to a control or test group by use of a random number table. Each mouse was then briefly anesthetized by isoflurane inhalation administered either by nose cone or by an instrument designed for small animal anesthesia, and an electronic ID chip was implanted by sterile syringe beneath the dorsal skin between the shoulder blades, after which the wound was closed by a drop of superglue (Loctite gel, purchased locally, or Nexaband S/C, purchased from Abbott Laboratories, North Chicago, IL, USA). UM and UT used chips purchased from AVID Microchip ID Systems (Catalog AVID3002; Folsom, LA, USA); TJL used chips purchased from Locus Technology (Manchester, MD, USA; catalog 1D‐100A). A portion of the distal tail (1 cm) was taken and frozen for later analysis of DNA polymorphisms, after which the mouse was permitted to awaken from the anesthesia. The duration of anesthesia was approximately 1–2 min.

Mice received Purina 5LG6 control diet until treatments were begun. Starting at various ages as listed in the results section, mice in the treatment groups received Purina 5LG6 containing the additives, at all three sites, and mice in the control group received Purina 5LG6.

Details of the methods used for health monitoring were provided in Miller *et al*. (2014); in brief, each of the three colonies was evaluated four times each year for infectious agents, including pinworm. All such tests were negative throughout the entire study period.

### Control and experimental diets

All diets were prepared by TestDiet, Inc., a division of Purina Mills (Richmond, IN, USA). Purina 5LG6 food containing each of the test substances, as well as batches of control diet, was prepared at intervals of approximately 4 months. Each batch of food was shipped at the same time to each of the three test sites. Protandim^®^ was a gift from LifeVantage Corp. (Sandy, UT, USA). It was mixed with chow at a concentration of 600 mg of Prot per kg of food (600 ppm) and fed to mice beginning at 10 months of age. The dose of Prot was increased from 600 ppm to 1200 ppm when the mice reached 17 months of age. 17aE2 was purchased from Steraloids Inc. (Newport, RI, USA) and mixed at a dose of 14.4 milligrams per kilogram diet (14.4 ppm). Mice were fed the 17aE2 diet continuously from 10 months of age. Metformin was a gift from Rafael de Cabo (National Institute on Aging Intramural Program) and used at a dose of 1000 mg kg^−1^ (0.1%) beginning at 9 months of age.

Microencapsulated rapamycin was obtained from Southwest Research Institute (San Antonio, TX) and mixed at a concentration of 14 ppm with metformin (1000 ppm) to determine the effects of the mixture on longevity. The mixture was fed to mice starting at 9 months of age. NDGA was purchased from Cayman Chemicals (Ann Arbor, MI, USA) and mixed at a concentration of either 800, 2500, or 5000 ppm and fed to male mice beginning at 4 months of age. A group of female mice received only the diet containing 5000 ppm of NDGA from 4 months of age. Ursodeoxycholic acid was obtained from HBC Chem, LLC (Union City, CA, USA) and fed beginning at 5 months of age at a dose of 5000 mg of UDCA per kg of food. Microencapsulated fish oil (containing DHA, minimum 132 mg/g of powder; and EPA plus DHA, minimum 168 mg kg^−1^ of powder) was purchased from Ocean Nutrition Canada (Dartmouth, NS, Canada), and incorporated into food at a concentration of 15 000 and 50 000 ppm. Mice were fed FO continuously from 9 months of age. Acarbose was purchased from Spectrum Chemical Mfg. Corp., Gardena, CA, USA. It was mixed at a concentration of 1000 mg of ACA per kilogram of diet (1000 ppm); mice were fed continuously from 16 months of age.

### Measurement of motor coordination and strength

Groups of male and female UM‐HET3 mice were fed control or NDGA‐containing diets (males – 2500 ppm; females – 5000 ppm) beginning at 13 months of age and were treated until they were 22 months of age. A group of 4‐month‐old mice fed the control diet served as young control group.

### Removal of mice from the longevity population

As described in detail in Miller *et al*. (2011), mice were removed from the study because of fighting, or accidental death, typically during chip implantation, or because of chip failure, or because they were used for another experimental purpose, such as testing for blood levels of a test agent. For survival analyses, all such mice were treated as alive at the date of their removal from the protocol, and lost to follow‐up thereafter. These mice were not included in calculations of median longevity.

### Estimation of age at death (lifespan)

Mice were examined at least daily for signs of ill health. Mice were euthanized for humane reasons if so severely moribund that they were considered, by an experienced technician, unlikely to survive for more than an additional 48 h. A mouse was considered severely moribund if it exhibited more than one of the following clinical signs: (i) inability to eat or to drink; (ii) severe lethargy, as indicated by reluctance to move when gently prodded with a forceps; (iii) severe balance or gait disturbance; (iv) rapid weight loss over a period of 1 week or more; or (v) an ulcerated or bleeding tumor. The age at which a moribund mouse was euthanized was taken as the best available estimate of its natural lifespan. Mice found dead were also noted at each daily inspection. At the time of analysis of the C2011 population, 954 female mice had died, 19 had been removed, and 11 were still alive, including one control and 10 mice in the MetRapa group. Among males, 1039 had died, 50 had been removed (mostly because of fighting), and 9 were still alive, including one control mice, one in the Prot group, one in the MetRapa group, and six in the 17aE2 group.

### Rotarod performance

Mice were tested on a rotarod (Rotamex‐5; Columbus Instruments, Columbus, Ohio) for 5 days by a technician who was blinded to the treatment groups. Testing on each day consisted of 8 trials with a 10‐min rest between trials 4 and 5. Each trial began with the rotarod set at an initial rate of 4 rpm, accelerating to a maximum 40 rpm within 300 s. The latency to fall was recorded by the Rotamex‐5 software, and the average latency to fall was calculated for each day.

### Grip strength

Grip strength was determined using a digital grip meter (Chatillion; Columbus Instruments), by a technician who was blinded to the experimental groups. Each animal was allowed to grip a wire mesh screen with either the forelimbs or forelimbs and hindlimbs. The tail was gently and steadily pulled to measure the maximum force and duration until the mouse released the wire mesh screen. The grip strength meter software collected the maximum grip force produced in gram‐force (gf) and the latency (milliseconds) to release the wire mesh screen for each of the 5 trials. The average grip strength and grip duration were calculated from the 5 trials.

### Measurement of 17aE2 estrogenicity

UM‐HET3 mice were ovariectomized at 2 months of age and then given mouse chow containing 17aE2 at 4.8 or 14.4 ppm or diet without hormone. Treatments continued for 2 months. Untreated intact mice (6.25 months of age at sacrifice) were also included as normal controls. Mice were euthanized and uteri were dissected free of fat and mesentery, nicked and blotted to release luminal fluid, and weighed.

### Statistical methods

For each sex, we performed site‐specific and combined site analysis. We calculated the Kaplan–Meier estimate of the median survival for the control group as well as for each treatment group. To compute the median percentage increase, we subtracted the median age in the control group from the corresponding value in the treatment group and divided the difference by median age of the control group and multiplied by 100. Using a two‐sided 5% significance level, we performed the log‐rank test to determine whether survival curves for mice receiving treatment differ from the survival function for control mice. Log‐rank tests that pooled data across the three test sites used a method that stratifies by site. To assess the maximum lifespan, we computed 90th percentile age of both the treated and control mice. To determine which treatments prolonged longevity in mice, we utilized the Wang–Allison test (Wang *et al*., 2004). This is the Fisher exact test comparing the numbers of mice surviving in control and treatment group at the age corresponding to the 90th percentile of lifespan in the joint survival distribution. To determine the difference between the survival function in treated and control group while controlling for site effect, we performed log‐rank test stratified by site. We further assessed the longevity of mice in all three sites combined using a modified version of the Wang–Allison test in the manner with which the 2 × 2 contingency table is constructed. Basically, we report the sum of corresponding site‐specific 2 × 2 tables cell entries as the combined site 2 × 2 table cell entries. This allows for information from all sites to be used in a balanced manner.

For statistical analysis of the rotarod performance, the average of time‐to‐fall sessions for each animal on each day (1–5) was considered as a repeated measure. We used a mixed‐effect linear model with a random intercept for males and females separately and estimated the main effects of day, group (Old Control, Old NDGA, and Young control), and the group‐by‐day interaction. The statistical significance of the group‐by‐day interaction would indicate that the groups have difference learning trajectories (as distinct from just being higher or lower on average). If the interaction term was not significant (*P* > 0.05), then the interaction was removed from the model. In order to reduce the number of comparisons, day was considered as a linear or a quadratic effect, depending on the significance of the quadratic term. We further compared each of the three subgroups to one another by excluding the other group for both males and females (Old Control vs. Young; Old Control vs. Old NDGA; Young vs. Old NDGA). In these models, we only considered the main effects of day (1–5) and group. We used r (v3.1+, Vienna, Austria) and the lmer^1^ and lmerTest ^2^ packages.

Grip strength data were analyzed by a one‐way ANOVA followed by the Tukey post hoc test to determine the significance of differences between means of individual groups.

## Author contributions

D.E.H., R.S., and R.A.M. are the principal investigators at the three collaborating institutions and are responsible for project design, supervision of technical personnel, interpretation of results, and preparation of manuscript drafts. J.F.N. and K.F. provided advice on experimental design and interpretation, and comments on the manuscript, and J.F.N. provided data on estrogenicity of 17aE2. Laboratory manager C.M.A. provided advice and supervised laboratory procedures and data collection at TJL. E.F supervised laboratory personnel and data collection at UT. M.A.J. supervised assays at the UT site. N.L.N. served as the project officer for the National Institute on Aging and contributed to program development, experimental design, and analysis. B.F.M., K.L.H, and J.M.M. proposed the Prot intervention; J.W.S. proposed 17aE2 for study; D.W.L. and D.M.S. proposed the Met intervention; D.E.H., K.F., A.R., and A.S. proposed combined treatment with Rapa and Met; A.A. and M.D. proposed UDCA; J.P.M., M.M., and G.E.R. suggested the FO intervention. J.N. and M.B. provided statistical support.

## Funding

National Institute on Aging, (Grant / Award Number: ‘AG013319’, ‘AG022303’, ‘AG022307’, ‘AG022308’, ‘AG024824’, ‘CA034196’).

## Conflict of interest

The University of Texas Health Science Center at San Antonio has applied for a patent, U.S. Patent Application No. 13/128,800, by inventors Zelton Dave Sharp and Randy Strong, for an encapsulated rapamycin formulation used in this paper. Under a licensing agreement between Rapamycin Holdings, Inc. and the University of Texas Health Science Center San Antonio, R. Strong and Z.D. Sharp, the University is entitled to milestone payments and royalty on sales of microencapsulated rapamycin. The university has a plan for managing conflict of interests under its ‘Policy and Procedures for Promoting Objectivity in Research by Managing, Reducing or Eliminating Conflicts of Interest’.

## Supporting information


**Fig. S1** Effects of 17aE2 on survival at each test site.
**Fig. S2** Dose‐dependent effects of 17aE2 on uterine weights in ovariectomized mice.
**Fig. S3** Effects of Prot on survival at each test site.
**Fig. S4** Effects of Met on survival at each test site.
**Fig. S5** Effects of Met/Rapa on survival at each test site.
**Fig. S6** Effects of ACA, initiated at 16 months, on survival at each test site.
**Fig. S7** Effects of 17aE2, Prot or UDCA on body weight in male and female mice.
**Fig. S8** Dose‐dependent effects of FO on body weight in male and female mice.

**Table S1** C2011 male and female mice, site‐specific results.
**Table S2** C2010 male and female mice, site‐specific results.
**Table S3** C2012 mice, site‐specific results for ACA started at 16 months.
**Table S4** Comparison of Met/Rapa to Historical Data for Rapa alone.
