# Supplementary material for: Longer lifespan in male mice treated with a weakly estrogenic agonist, an antioxidant, an α‐glucosidase inhibitor or a Nrf2‐inducer
Source: Aging Cell. 2016 Jun 16;15(5):872–84. doi: 10.1111/acel.12496 (PMC5013015; doi:10.1111/acel.12496)
Supplement: Supplementary file 1 — Fig. S1 Effects of 17aE2 on survival at each test site. Fig. S2 Dose‐dependent effects of 17aE2 on uterine weights in ovariectomized mice. Fig. S3 Effects of Prot on survival at each test site. Fig. S4 Effects of Met on survival at each test site. Fig. S5 Effects of Met/Rapa on survival at each test site. Fig. S6 Effects of ACA, initiated at 16 months, on survival at each test site. Fig. S7 Effects of 17aE2, Prot or UDCA on body weight in male and female mice. Fig. S8 Dose‐dependent effects of FO on body weight in male and female mice. [file ACEL-15-872-s001.docx]

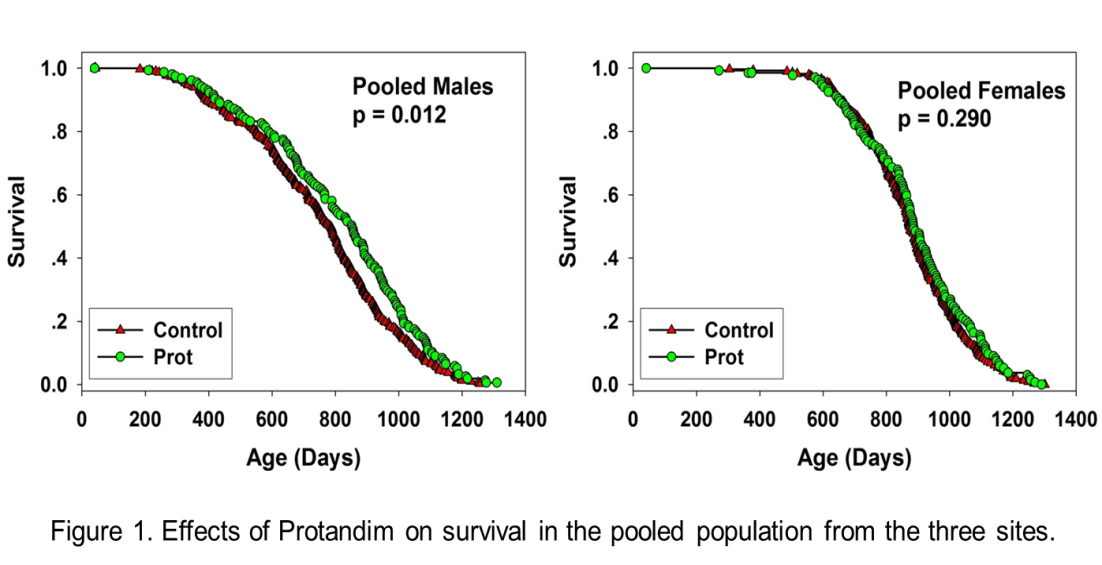

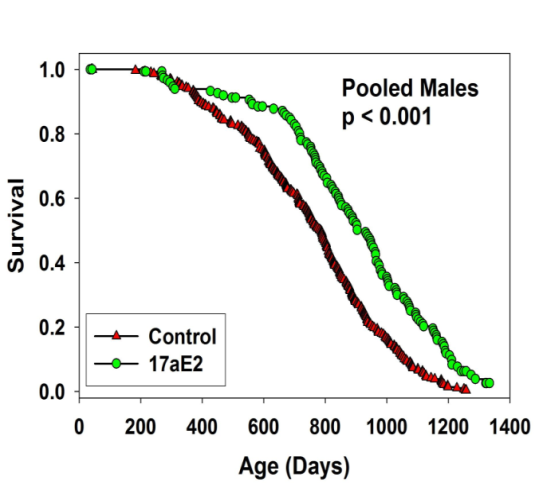

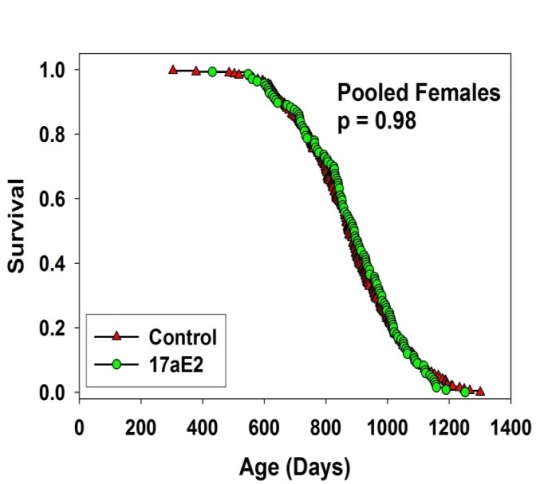


B

A

C

D

Figure 1.


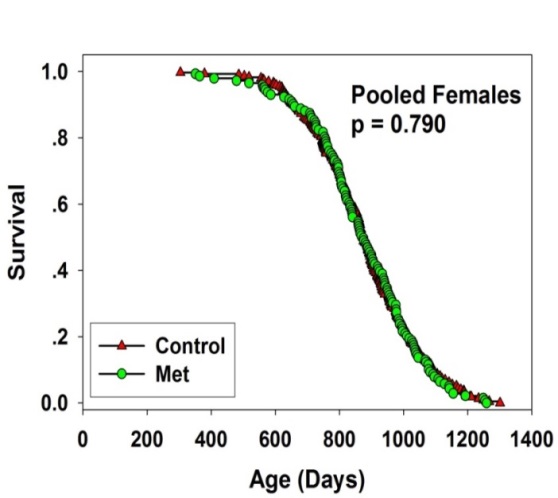

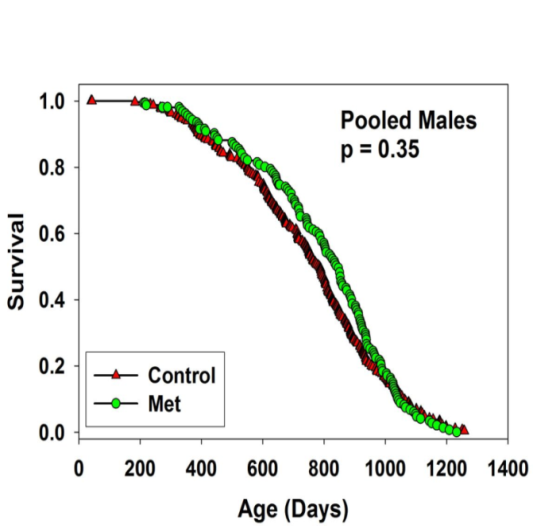

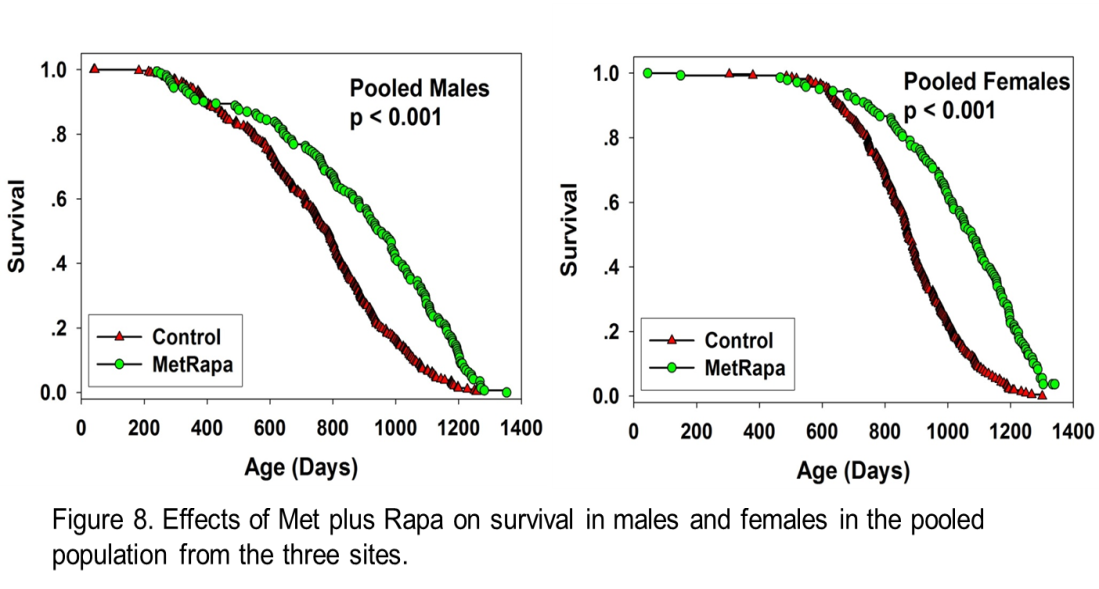


B

A

C

D

Figure 2.


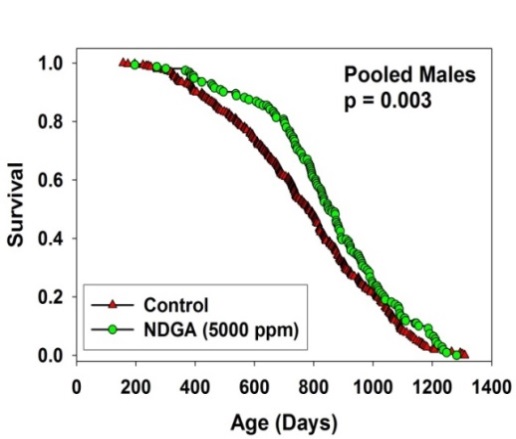

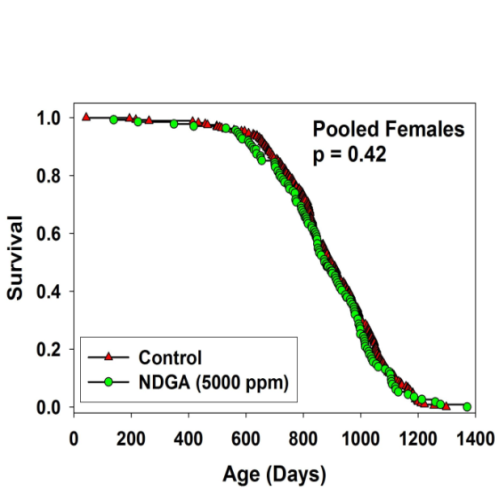

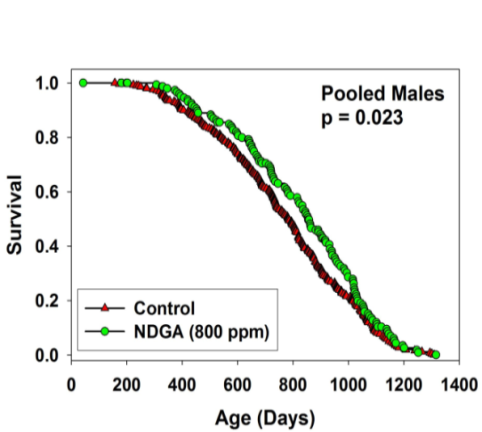

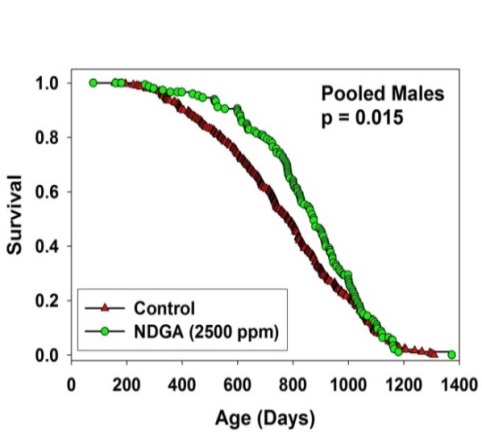


B

A

C

D

Figure 3


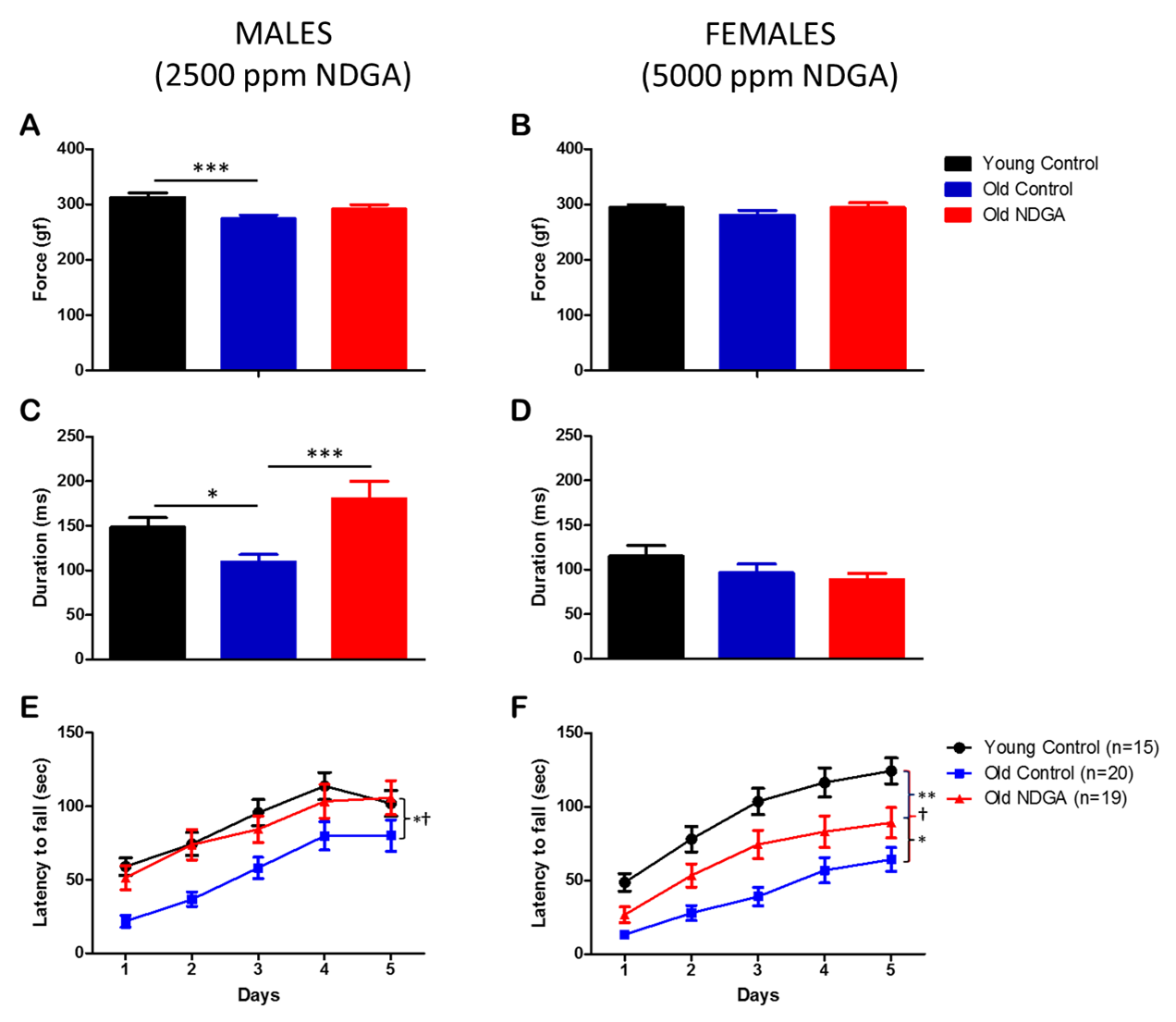


Figure 4


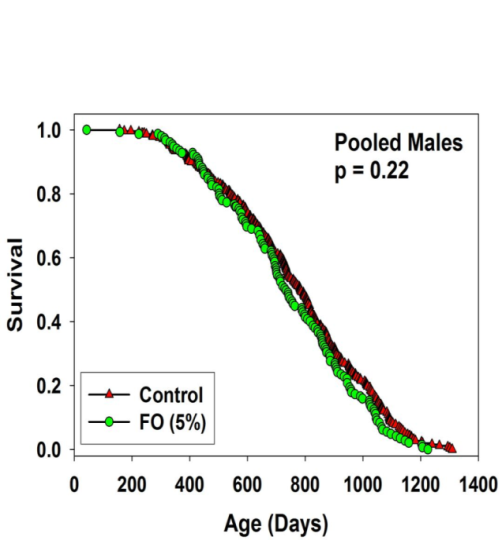

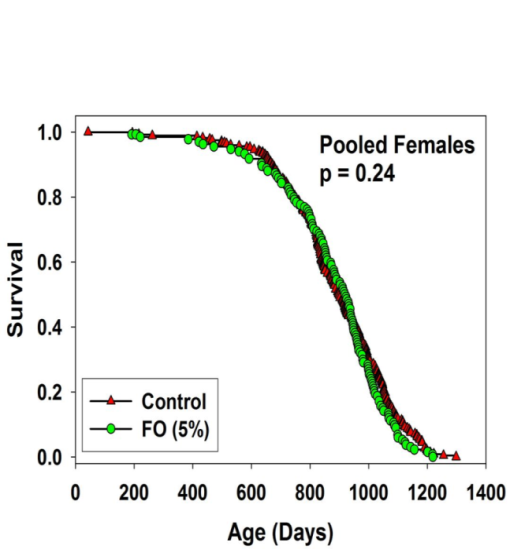

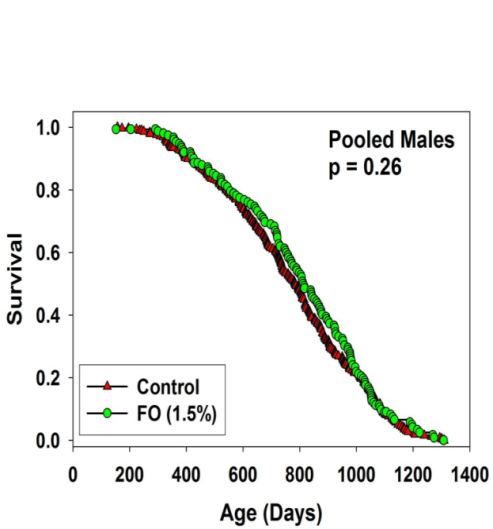

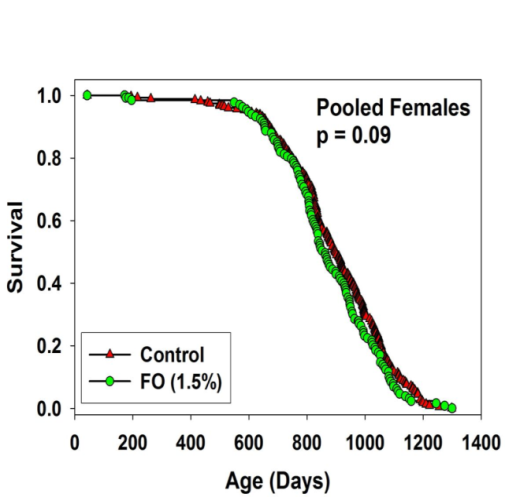


B

A

C

D

Figure 5


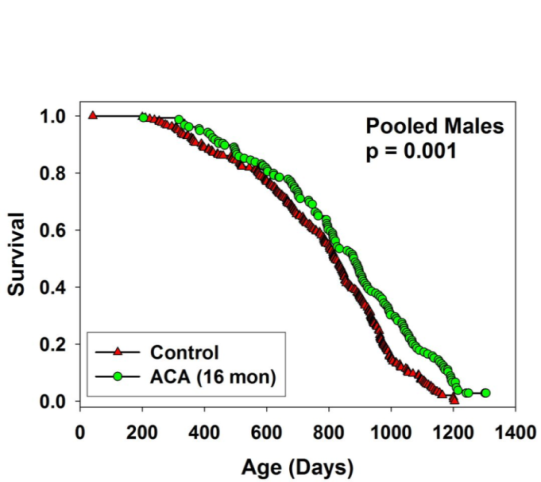

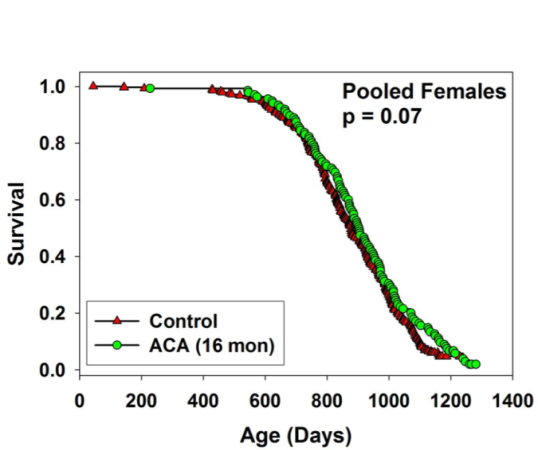

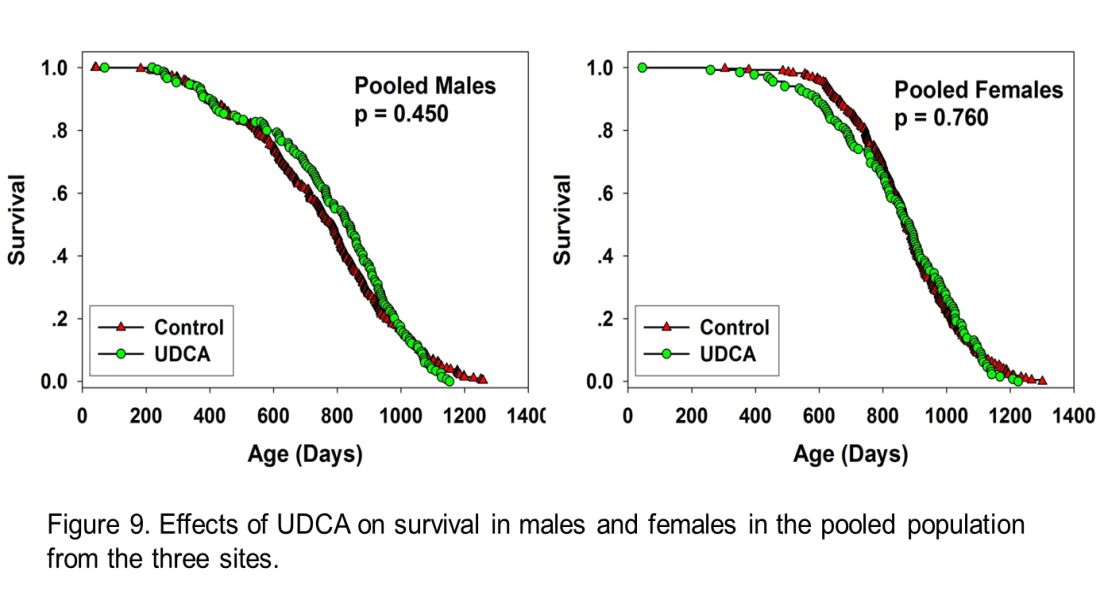


B

A

C

D

Figure 6

Supplemental Figure 1


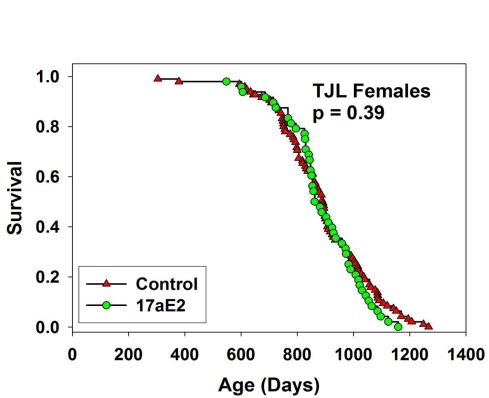

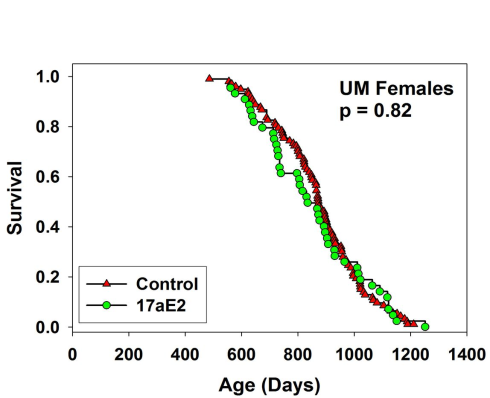

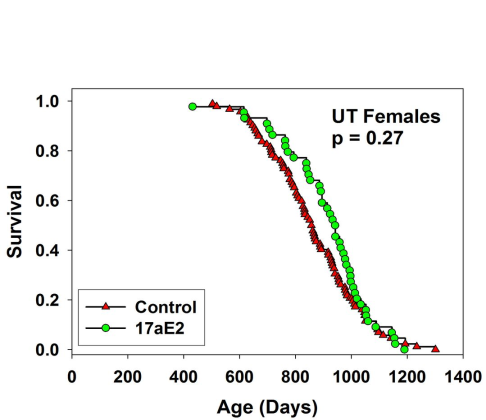

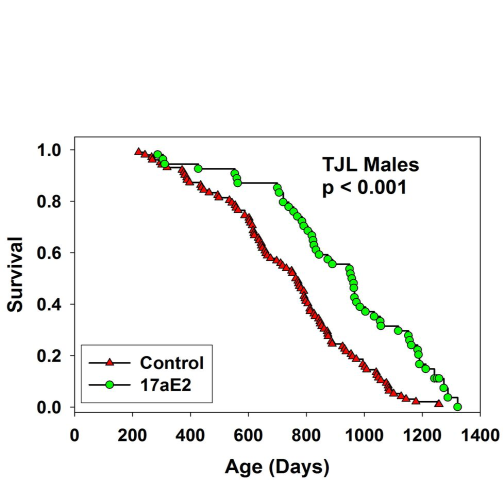

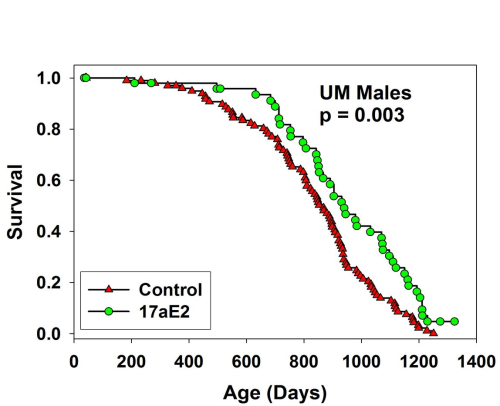

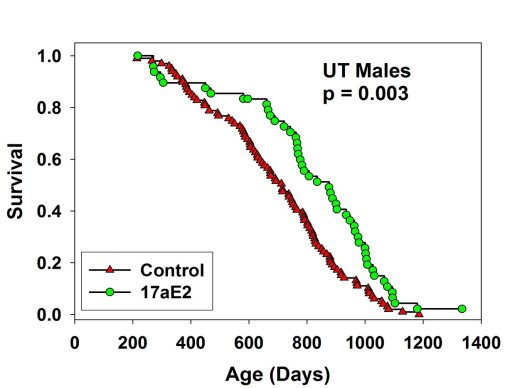


B

A

C

E

D

F

Supplemental Figure 2

Supplemental Figure 3


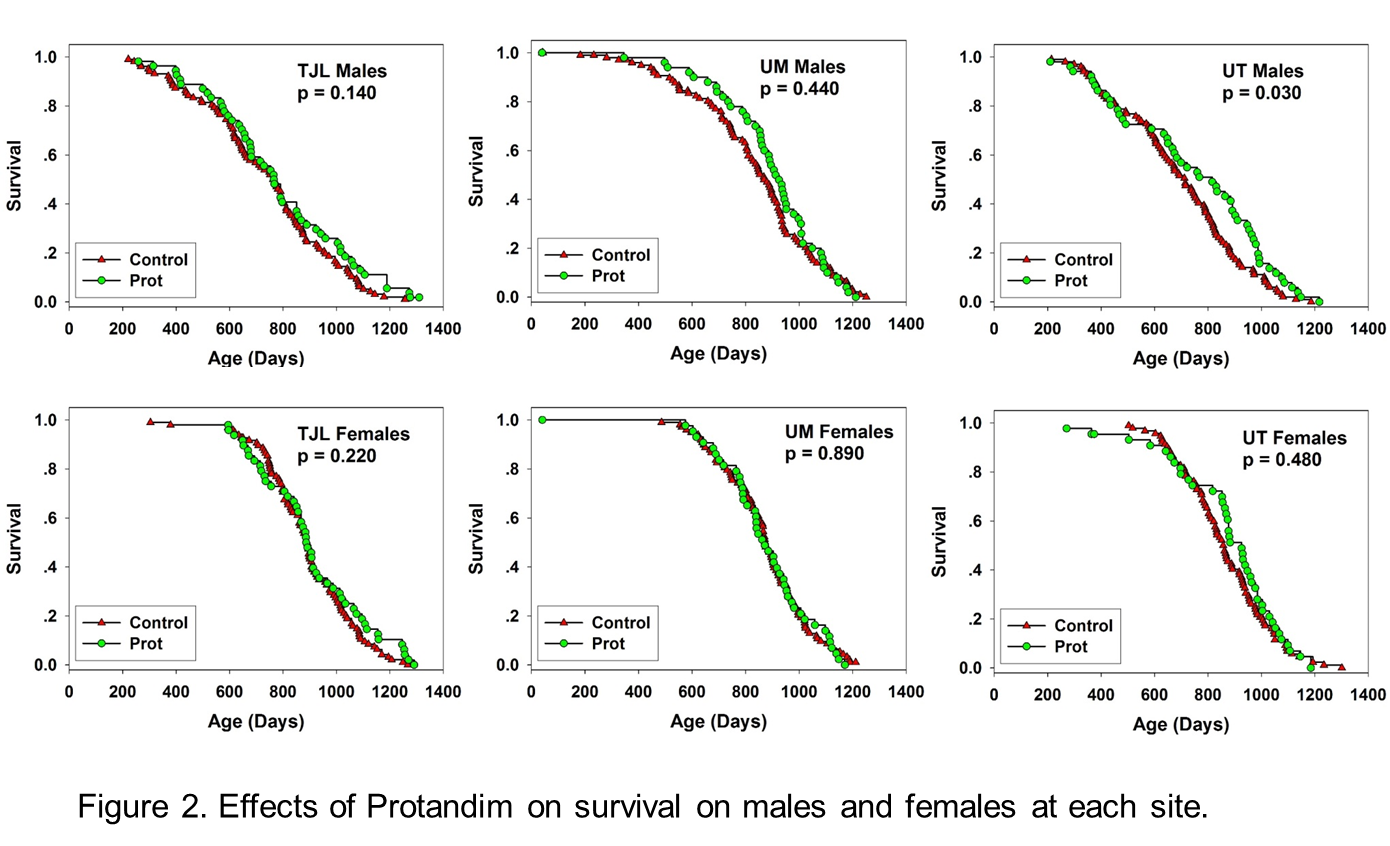


B

A

C

E

D

F

Supplemental Figure 4


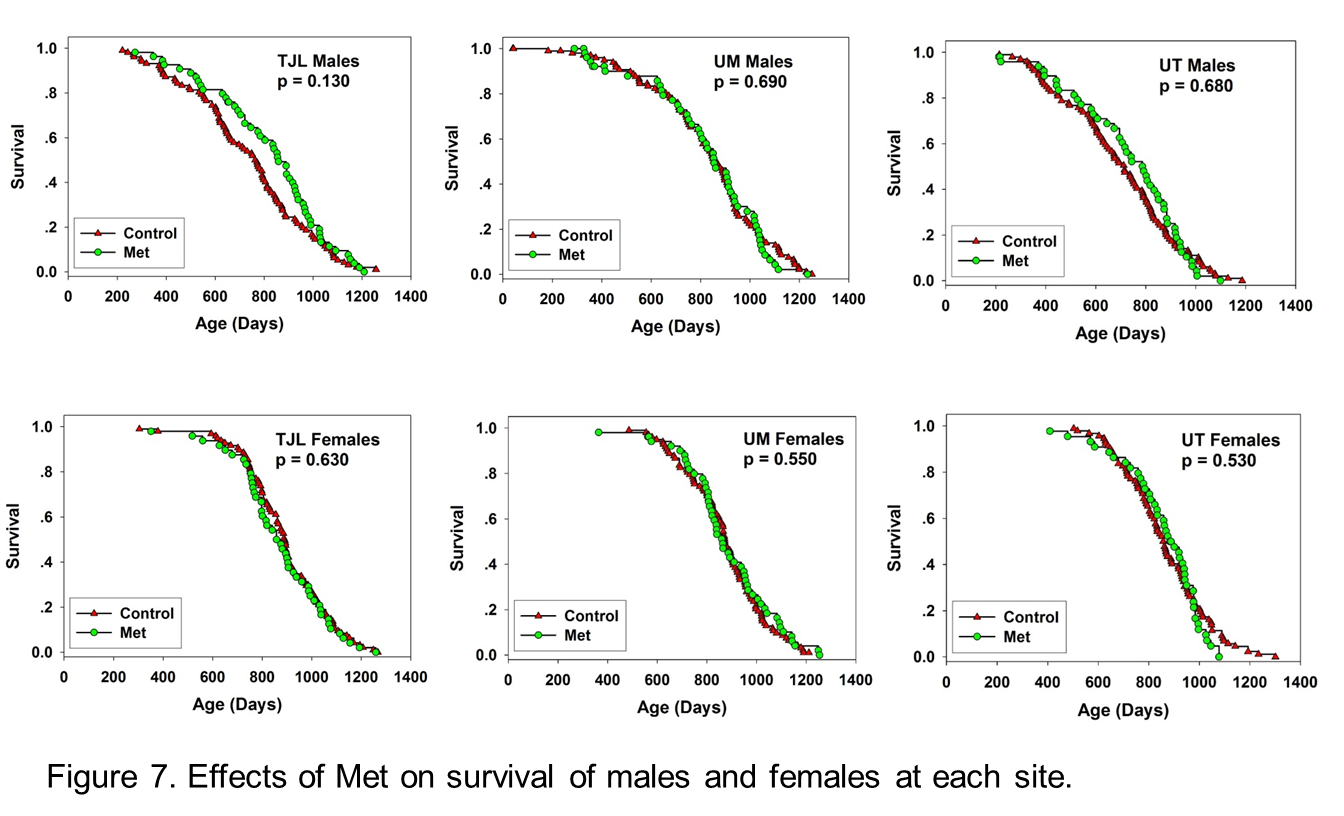


B

A

C

E

D

F

Supplemental Figure 5


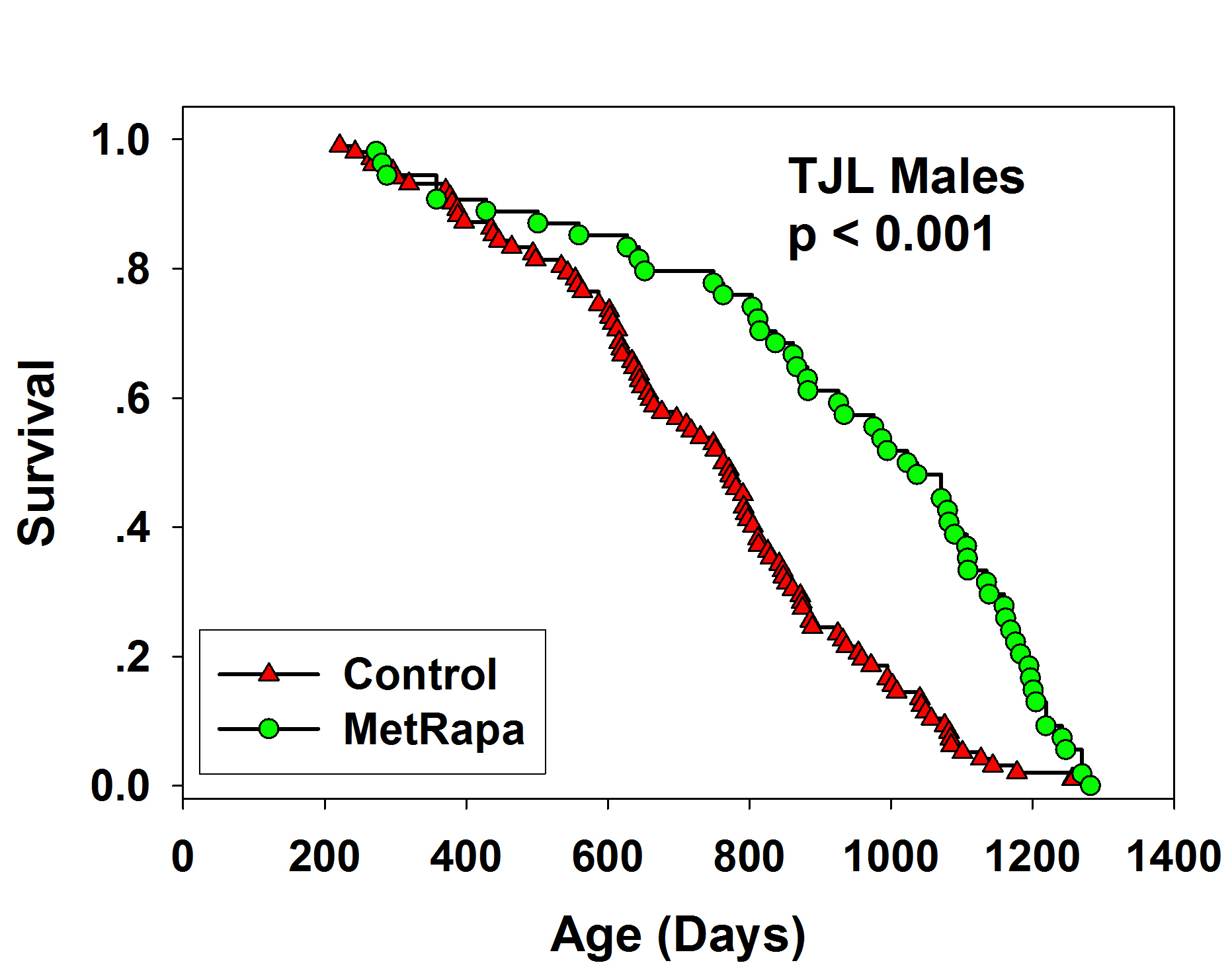

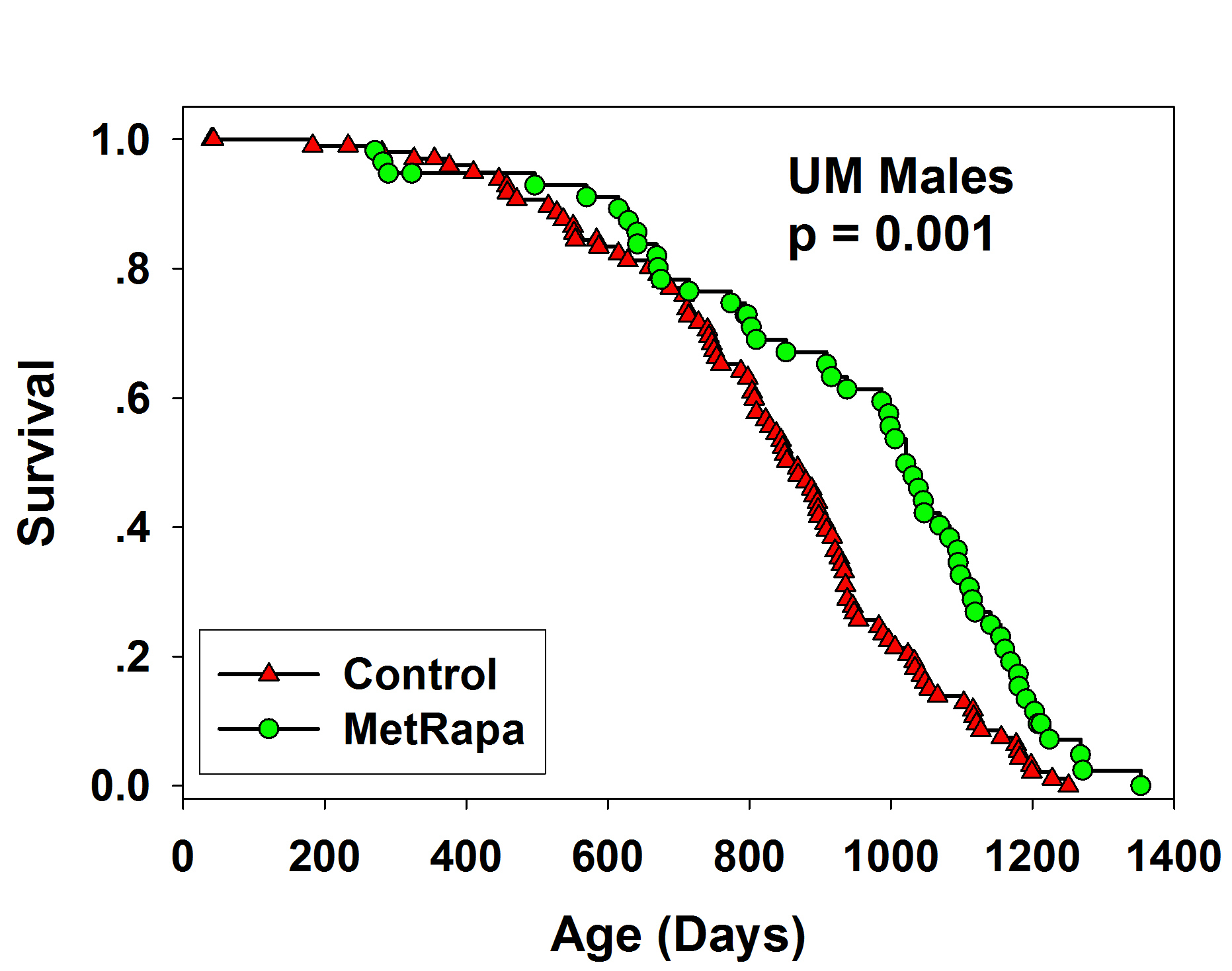

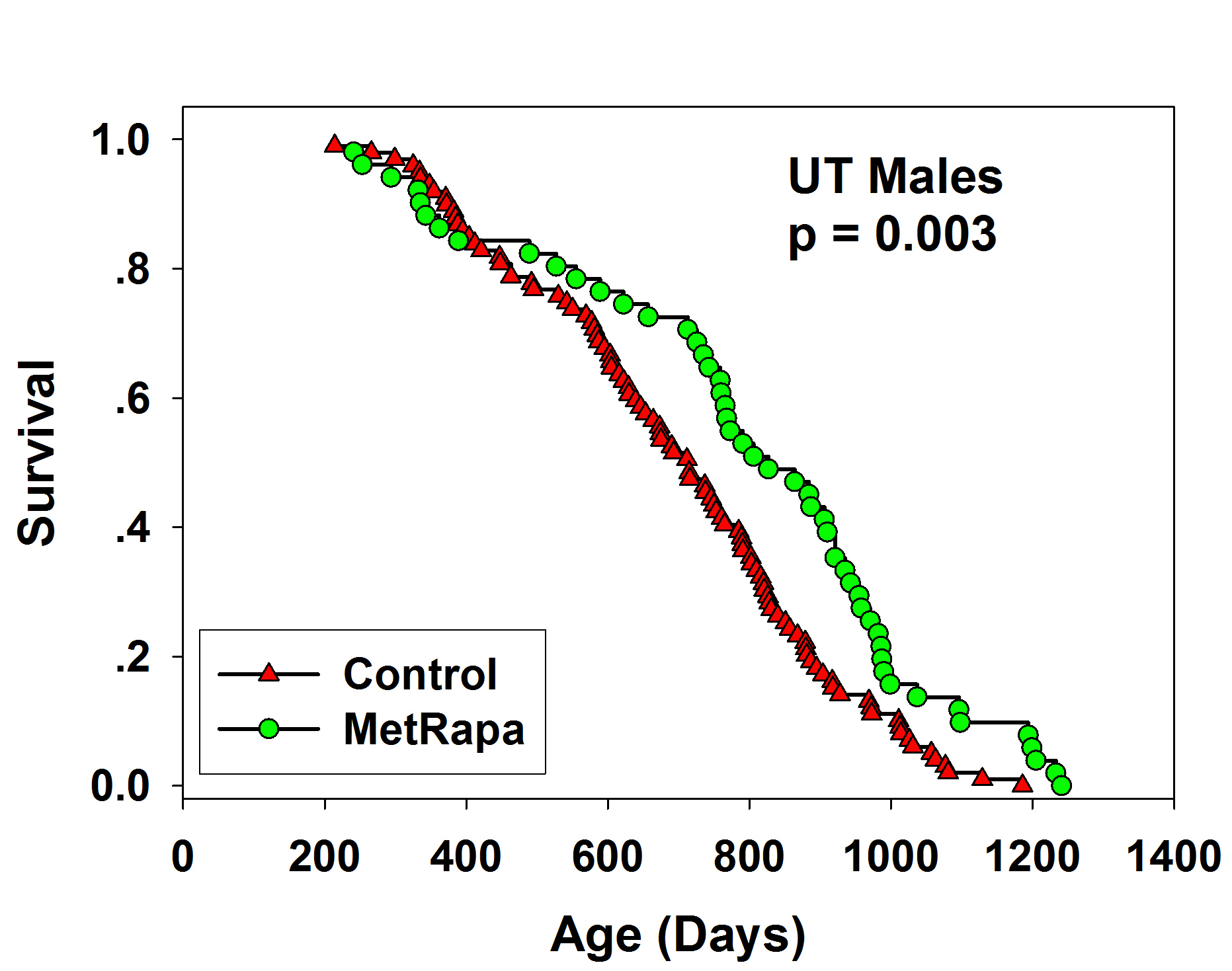

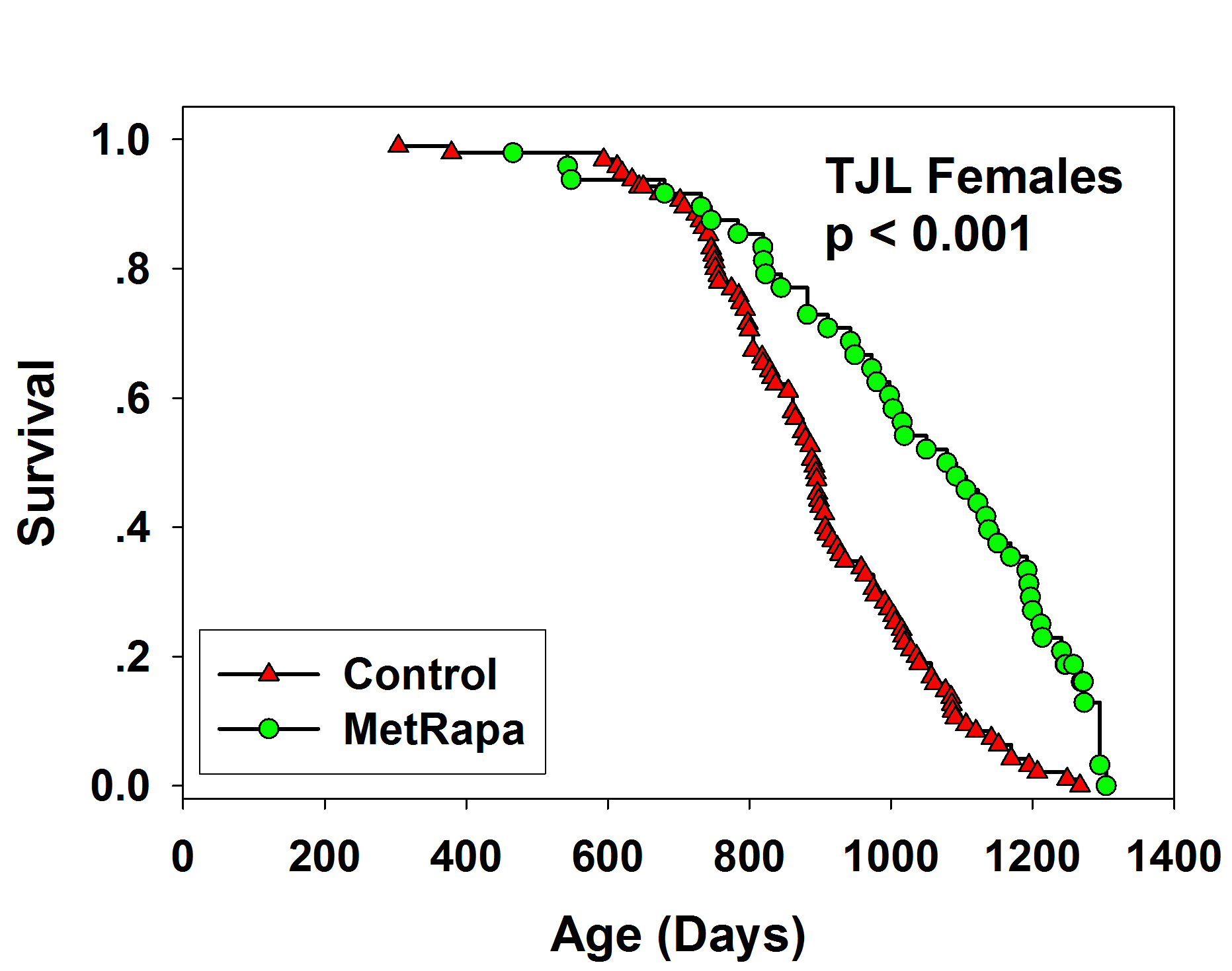

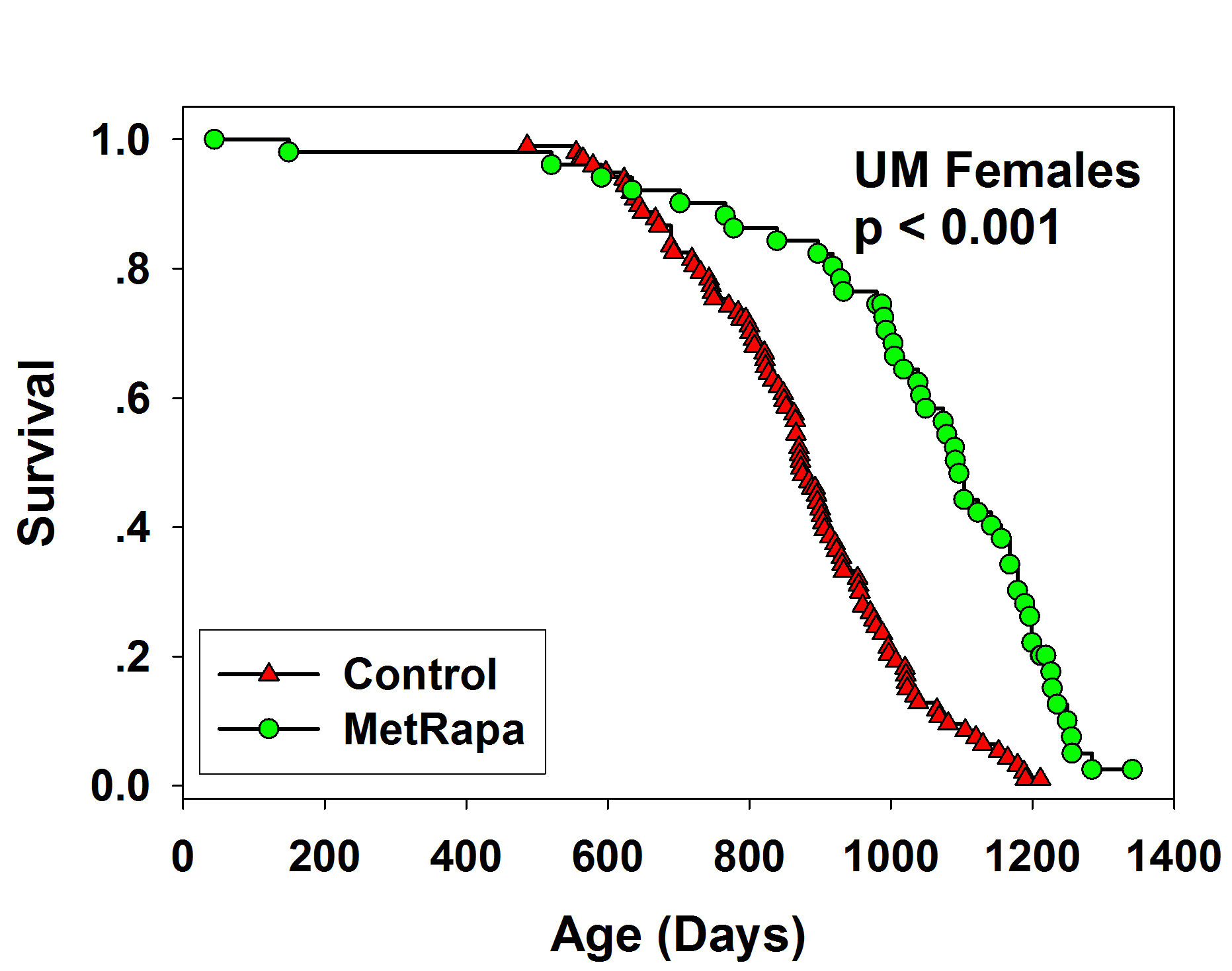

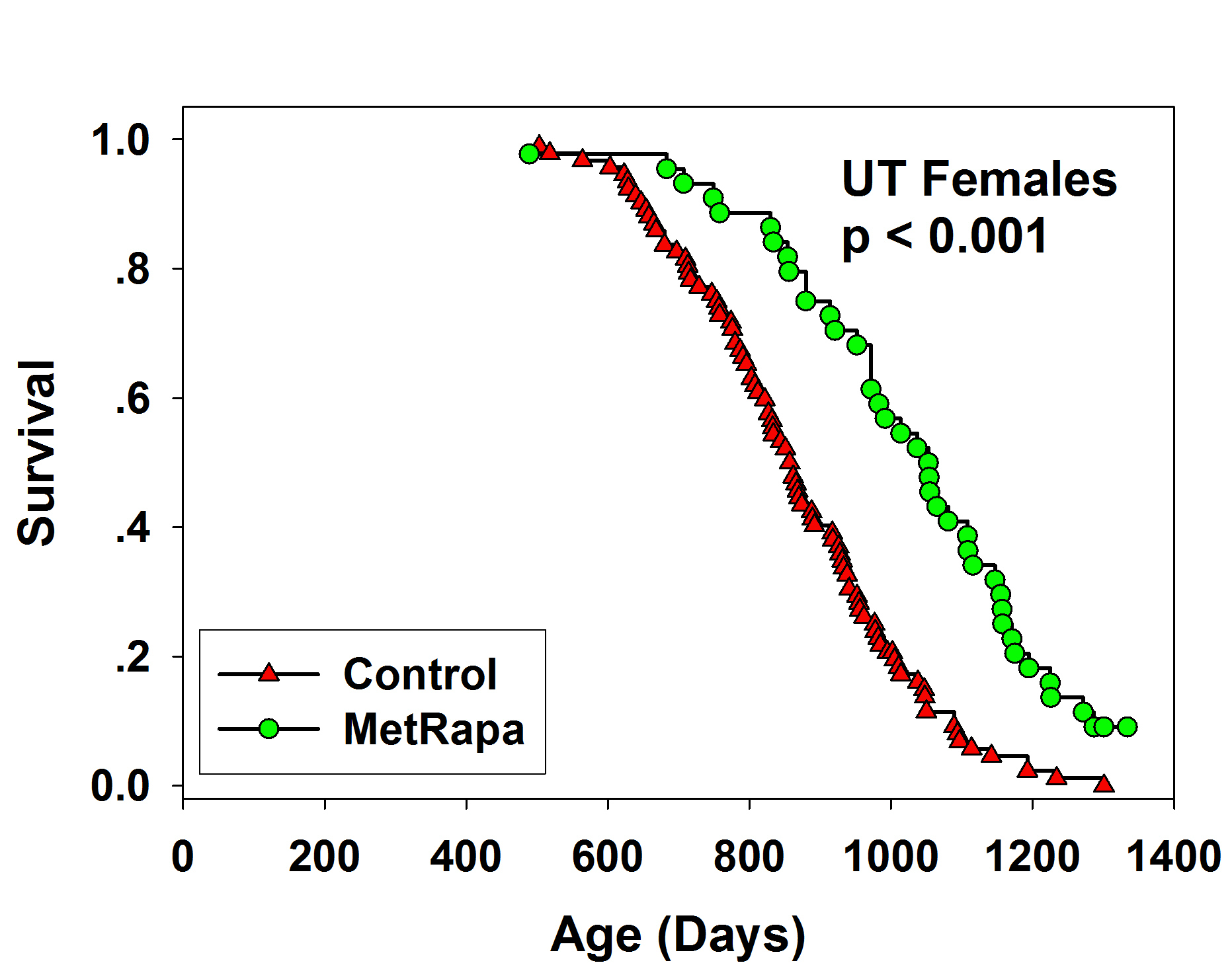


B

A

C

E

D

F

Supplemental Figure 6


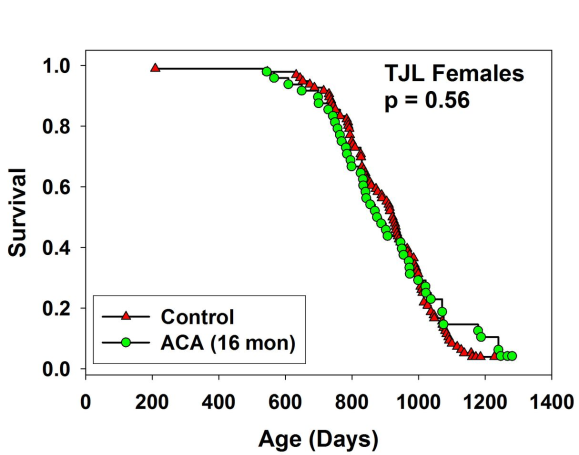

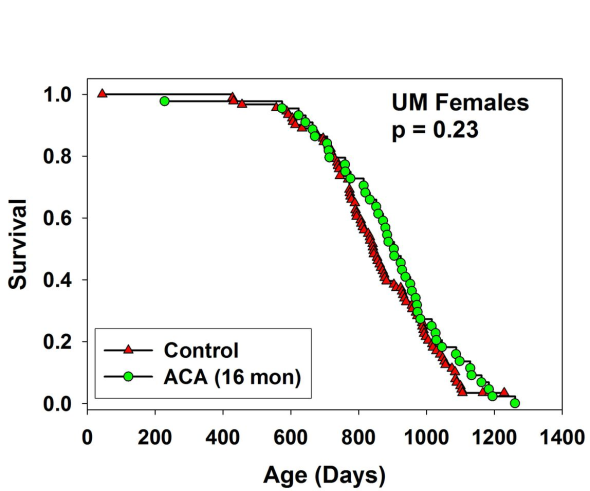

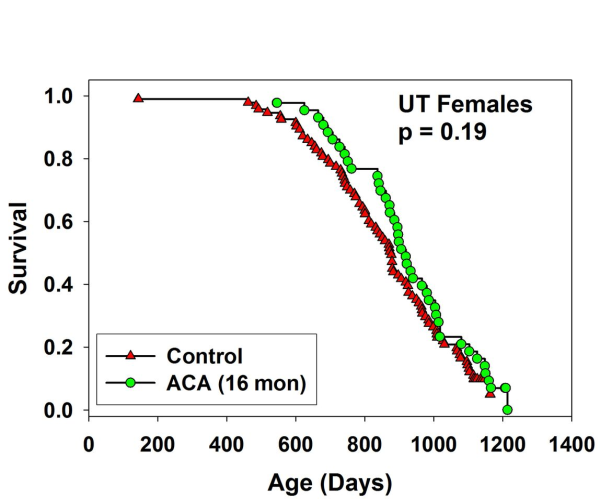

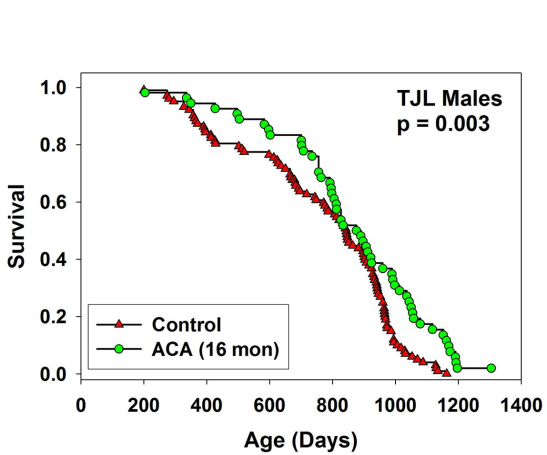

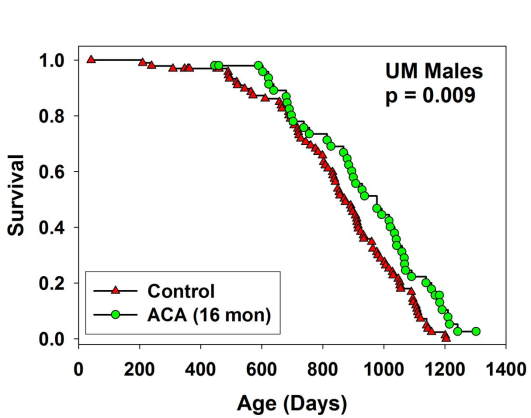

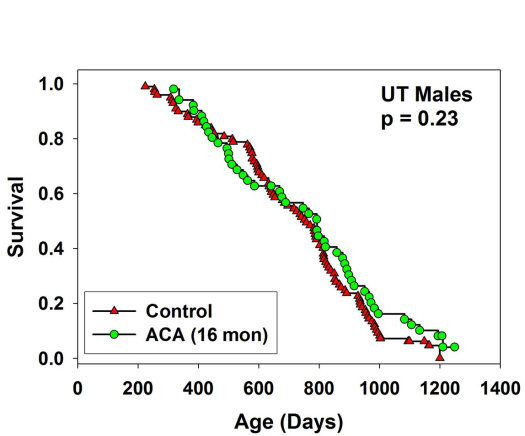


B

A

C

E

D

F

Supplemental Figure 7


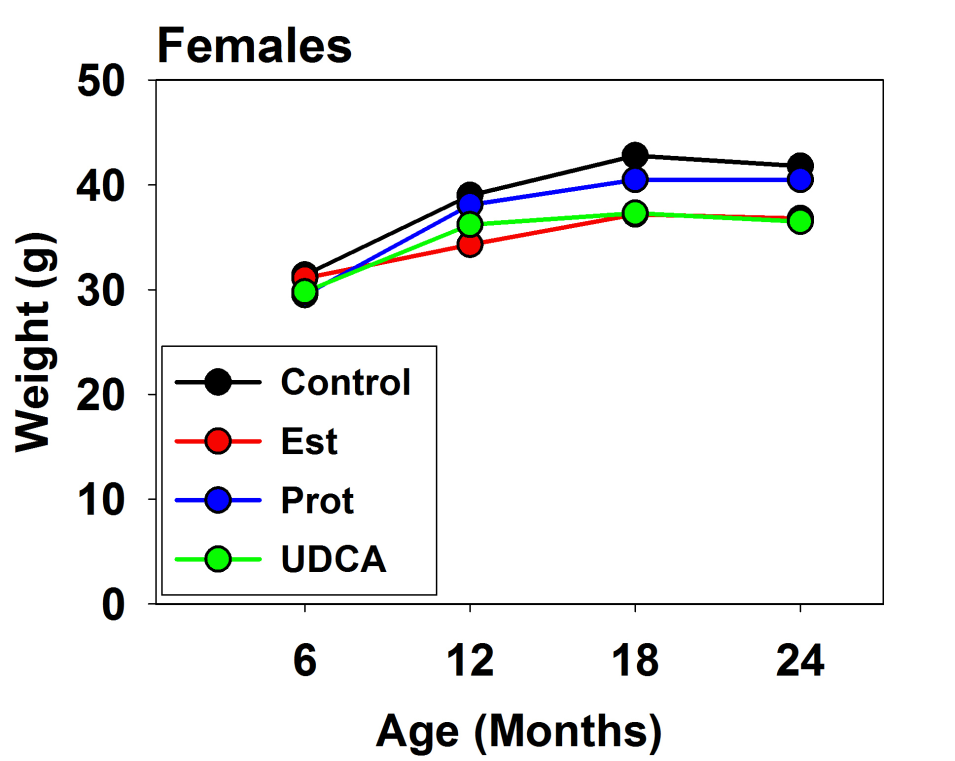

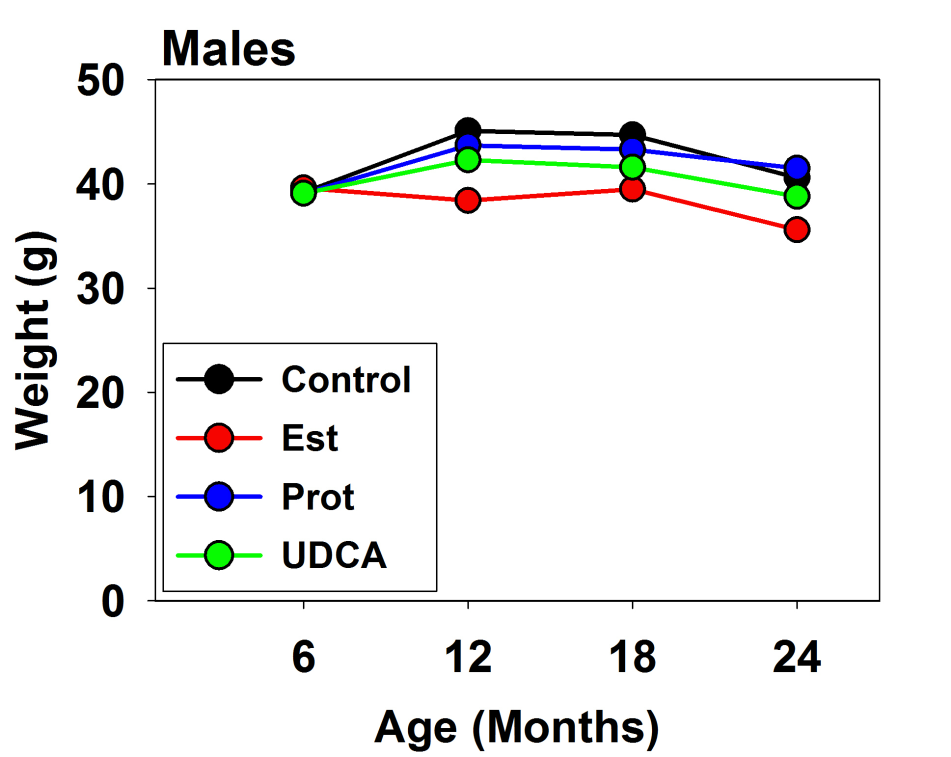


A

B

Supplemental Figure 8


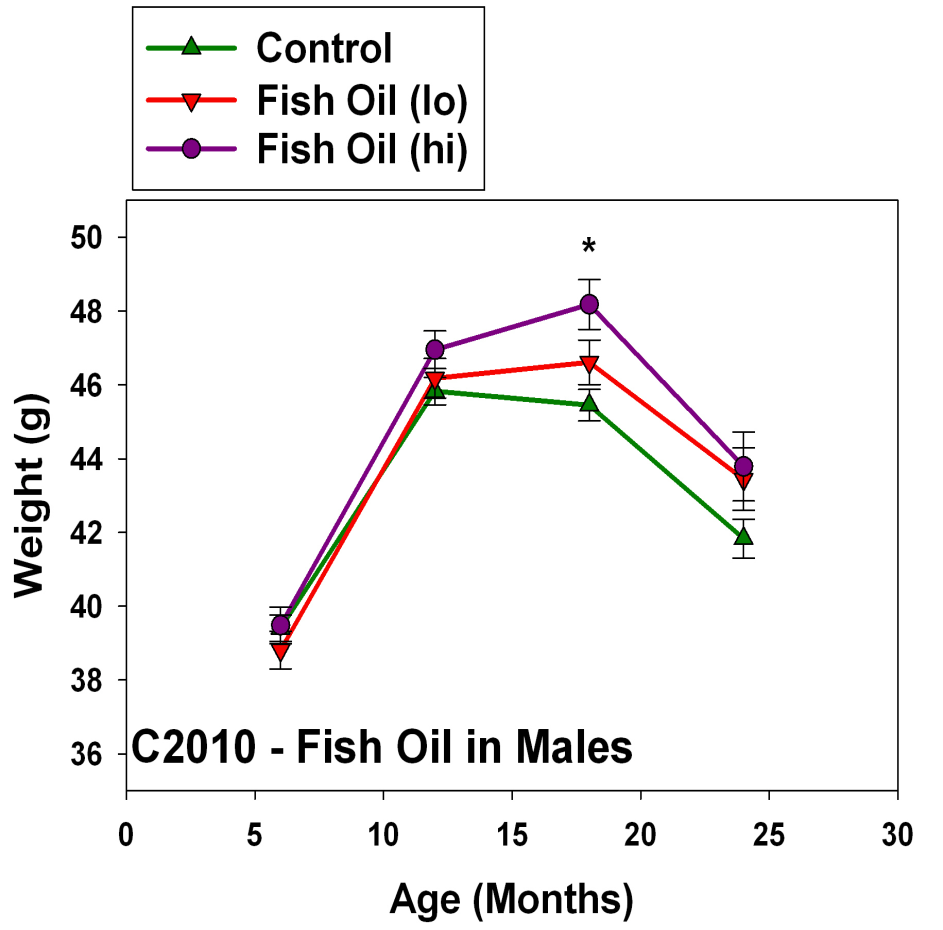

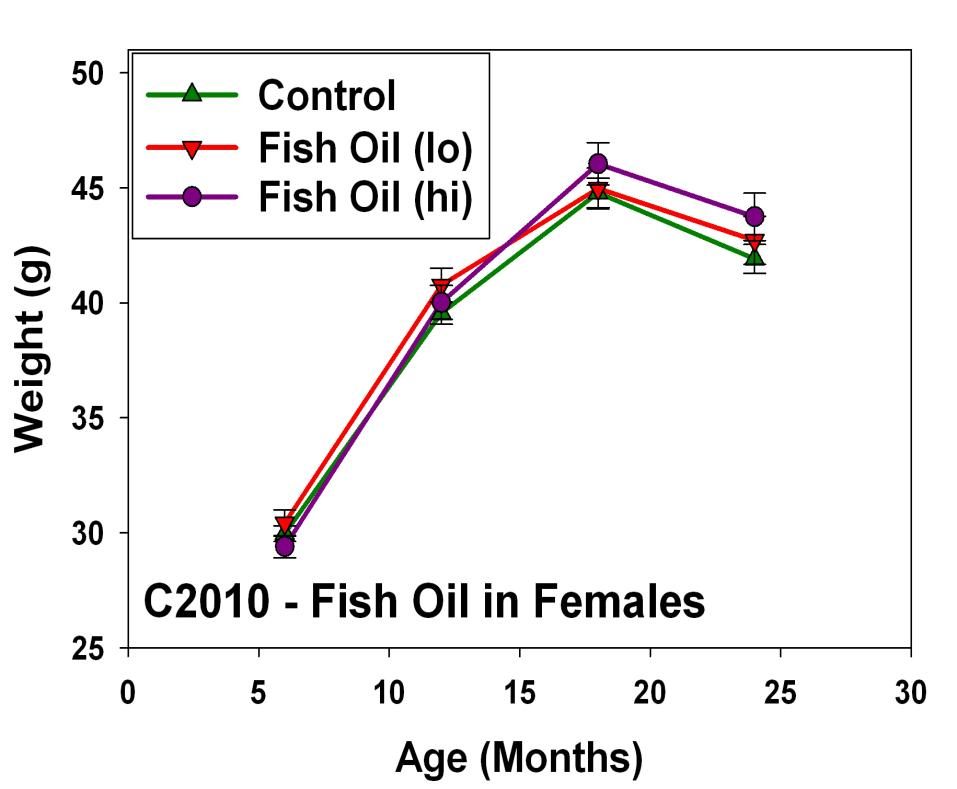


A

B

**Fig. S1.** Effects of 17aE2 on survival at each test site. Left to right: TJL, UM, UT. (A–C) males. (D–F) females. *P*-values calculated by the log-rank test. See Supplemental Table  1 for statistical results.

**Fig. S2.** Dose-dependent effects of 17aE2 on uterine weights in ovariectomized mice. 2 month old mice were ovariectomized (OVX) and treated with diet containing either 4.8 or 14.4 ppm 17aE2. Uterine weights were taken at sacrifice 2 months after start of treatment. Untreated intact mice (used as a positive control group) were 6.25 months old at sacrifice. Data are expressed as the mean ± SEM of 4 to 10 mice per group. **, significantly different from OVX control group, p = 0.0015. ***, significantly different from OVX control group, p < 0.001.

**Fig. S3.** Effects of Prot on survival at each test site. Left to right: TJL, UM, UT. A-C: males. D-F: females. P-values calculated by the log-rank test. See Supplemental Table  1 for statistical results.

**Fig. S4.** Effects of Met on survival at each test site. Left to right: TJL, UM, UT. A-C: males. D-F: females. P-values calculated by the log-rank test. See Supplemental Table  1 for statistical results.

**Fig. S5.** Effects of Met/Rapa on survival at each test site. Left to right: TJL, UM, UT. A-C: males. D-F: females. P-values calculated by the log-rank test. See Supplemental Table  1 for statistical results.

**Fig. S6.** Effects of ACA, initiated at 16 months, on survival at each test site. Left to right: TJL, UM, UT. A-C: males. D-F: females. P-values calculated by the log-rank test. See Supplemental Table 3 for statistical results.

**Fig. S7.** Effects of 17aE2, Prot or UDCA on body weight in male and female mice. A: males. B: females.

**Fig. S8.** Dose-dependent effects of FO on body weight in male and female mice. A: males. B: females.
