# Supplementary material for: Longer lifespan in male mice treated with a weakly estrogenic agonist, an antioxidant, an α‐glucosidase inhibitor or a Nrf2‐inducer
Source: Aging Cell. 2016 Jun 16;15(5):872–84. doi: 10.1111/acel.12496 (PMC5013015; doi:10.1111/acel.12496)
Supplement: Supplementary file 2 — Table S1 C2011 male and female mice, site‐specific results. Table S2 C2010 male and female mice, site‐specific results. Table S3 C2012 mice, site‐specific results for ACA started at 16 months. Table S4 Comparison of Met/Rapa to Historical Data for Rapa alone. [file ACEL-15-872-s002.docx]

Supplemental Table 1: C2011 Male Mice, site-specific results

| Males: TJL | | |  |  |  |  |  |
| --- | --- | --- | --- | --- | --- | --- | --- |
| Group | Number | Median  Days | Median % Increase | Log-rank  P-value | 90^th^ %ile Days | 90th %ile Increase | Wang-Allison P-value |
| Control | 101 | 763 |  |  | 1057 |  |  |
| 17aE2 | 54 | 960 | 26 | 0.000 | 1274 | 21 | 0.000 |
| Prot | 54 | 767 | 1 | 0.144 | 1189 | 12 | 0.267 |
| Met | 53 | 859 | 13 | 0.128 | 1093 | 3 | 0.786 |
| Met/Rapa | 54 | 1030 | 35 | 0.000 | 1219 | 15 | 0.000 |
| UDCA | 54 | 863.5 | 13 | 0.596 | 1067 | 1 | 0.789 |
| Males:UM | | |  |  |  |  |  |
| Group | Number | Median  Days | Median % Increase | Log-rank  P-value | 90th %ile Days | 90th %ile Increase | Wang-Allison P-value |
| Control | 94 | 861 |  |  | 1121 |  |  |
| 17aE2 | 43 | 940 | 9 | 0.003 | 1211 | 8 | 0.037 |
| Prot | 50 | 919 | 7 | 0.439 | 1123 | 0 | 1.000 |
| Met | 47 | 855 | -1 | 0.687 | 1061 | -5 | 0.142 |
| Met/Rapa | 53 | 1021 | 19 | 0.000 | 1208 | 8 | 0.037 |
| UDCA | 44 | 844 | -2 | 0.459 | 1088 | -3 | 0.548 |
| Males: UT | | |  |  |  |  |  |
| Group | Number | Median  Days | Median % Increase | Log-rank  P-value | 90th %ile Days | 90th %ile Increase | Wang-Allison P-value |
| Control | 99 | 715 |  |  | 1013 |  |  |
| 17aE2 | 47 | 876 | 23 | 0.003 | 1094 | 8 | 0.082 |
| Prot | 51 | 816 | 14 | 0.034 | 1077 | 6 | 0.148 |
| Met | 48 | 790 | 10 | 0.681 | 985 | -3 | 0.774 |
| Met/Rapa | 51 | 827 | 16 | 0.003 | 1098 | 8 | 0.148 |
| UDCA | 51 | 789 | 10 | 0.164 | 1034 | 2 | 0.389 |

Supplemental Table 1, continued: C2011 Female Mice, site-specific results

| Females: TJL | | |  |  |  |  |  |
| --- | --- | --- | --- | --- | --- | --- | --- |
| Group | Number | Median  Days | Median % Increase | Log-rank  P-value | 90^th^ %ile  Days | 90th %ile Increase | Wang-Allison P-value |
| Control | 95 | 892 |  |  | 1106 |  |  |
| 17aE2 | 48 | 872 | -2 | 0.389 | 1065 | -4 | 0.386 |
| Prot | 48 | 890 | 0 | 0.220 | 1246 | 13 | 0.263 |
| Met | 48 | 867 | -3 | 0.630 | 1113 | 1 | 1.000 |
| Met/Rapa | 48 | 1086 | 22 | 0.000 | 1295 | 17 | 0.000 |
| UDCA | 47 | 913 | 2 | 0.694 | 1107 | 0 | 1.000 |
| Females: UM | | |  |  |  |  |  |
| Group | Number | Median  Days | Median % Increase | Log-rank  P-value | 90^th^ %ile Days | 90th %ile Increase | Wang-Allison P-value |
| Control | 95 | 872 |  |  | 1081 |  |  |
| 17aE2 | 43 | 835 | -4 | 0.821 | 1122 | 4 | 0.366 |
| Prot | 43 | 872 | 0 | 0.894 | 1116 | 3 | 0.366 |
| Met | 49 | 864 | -1 | 0.551 | 1142 | 6 | 0.580 |
| Met/Rapa | 50 | 1094 | 25 | 0.000 | 1252 | 16 | 0.000 |
|  |  |  |  |  |  |  |  |
| UDCA | 42 | 819 | -6 | 0.498 | 1110 | 3 | 0.761 |
| Females: UT | | |  |  |  |  |  |
| Group | Number | Median  Days | Median % Increase | Log-rank  P-value | 90^th^ %ile Days | 90th %ile Increase | Wang-Allison P-value |
| Control | 91 | 857 |  |  | 1089 |  |  |
| 17aE2 | 44 | 943 | 10 | 0.272 | 1087 | 0 | 1.000 |
| Prot | 43 | 926 | 8 | 0.483 | 1099 | 1 | 0.755 |
| Met | 43 | 884 | 3 | 0.533 | 1026 | -6 | 0.225 |
| Met/Rapa | 44 | 1054 | 23 | 0.000 | 1287 | 18 | 0.002 |
| UDCA | 44 | 863 | 1 | 0.778 | 1097 | 1 | 0.384 |

Supplemental Table 2: C2010 Male Mice, site-specific results

| Males: TJL | | |  |  |  |  |  |
| --- | --- | --- | --- | --- | --- | --- | --- |
| Group | Number | Median  Days | Median % Increase | Log-rank  P-value | 90th %ile  Days | 90th %ile Increase | Wang-Allison P-value |
| Control | 91 | 683 |  |  | 1067 |  |  |
| FO(50000) | 51 | 789 | 16 | 0.445 | 1025 | -4 | 0.57 |
| FO(15000) | 50 | 737 | 8 | 0.853 | 1046 | -2 | 0.77 |
| NDGA(5000) | 45 | 812 | 19 | 0.072 | 1020 | -4 | 0.39 |
| NDGA(2500 | 42 | 794 | 16 | 0.080 | 1034 | -3 | 0.55 |
| NDGA(800) | 39 | 731 | 7 | 0.364 | 999 | -6 | 0.34 |
| Males:UM | | |  |  |  |  |  |
| Group | Number | Median  Days | Median % Increase | Log-rank  P-value | 90th %ile  Days | 90th %ile Increase | Wang-Allison P-value |
| Control | 88 | 926 |  |  | 1146 |  |  |
| FO(50000) | 44 | 754 | -19 | 0.003 | 1058 | -8 | 0.38 |
| FO(15000) | 43 | 951 | 3 | 0.815 | 1061 | -7 | 1.00 |
| NDGA(5000) | 48 | 864 | -7 | 0.869 | 1210 | 6 | 0.03 |
| NDGA(2500 | 44 | 945 | 2 | 0.709 | 1105 | -4 | 0.77 |
| NDGA(800) | 46 | 992 | 7 | 0.265 | 1164 | 2 | 1.00 |
| Males: UT | | |  |  |  |  |  |
| Group | Number | Median  Days | Median % Increase | Log-rank  P-value | 90th %ile  Days | 90th %ile Increase | Wang-Allison P-value |
| Control | 95 | 732 |  |  | 1041 |  |  |
| FO(50000) | 51 | 703 | -4 | 0.721 | 1038 | 0 | 1.00 |
| FO(15000) | 48 | 801 | 9 | 0.055 | 1128 | 8 | 0.26 |
| NDGA(5000) | 44 | 840 | 15 | 0.002 | 1094 | 5 | 0.14 |
| NDGA(2500 | 47 | 816 | 11 | 0.041 | 1102 | 6 | 0.57 |
| NDGA(800) | 47 | 771 | 5 | 0.059 | 1145 | 10 | 0.57 |

Supplemental Table 2, continued: C2010 Female Mice, site-specific results

| Females: TJL | | |  |  |  |  |  |
| --- | --- | --- | --- | --- | --- | --- | --- |
| Group | Number | Median  Days | Median % Increase | Log-rank  P-value | 90th %ile  Days | 90th %ile Increase | Wang-Allison P-value |
| Control | 92 | 864 |  |  | 1102 |  |  |
| FO(50000) | 48 | 918 | 6 | 0.920 | 1085 | -2 | 0.77 |
| FO(15000) | 47 | 852 | -1 | 0.495 | 1053 | -4 | 0.77 |
| NDGA(5000) | 44 | 859 | -1 | 0.887 | 1106 | 0 | 0.77 |
| Females: UM | | |  |  |  |  |  |
| Group | Number | Median  Days | Median % Increase | Log-rank  P-value | 90th %ile  Days | 90th %ile Increase | Wang-Allison P-value |
| Control | 88 | 931 |  |  | 1132 |  |  |
| FO(50000) | 43 | 947 | 2 | 0.742 | 1100 | -3 | 1.00 |
| FO(15000) | 41 | 873 | -6 | 0.106 | 1089 | -4 | 0.22 |
| NDGA(5000) | 41 | 947 | 2 | 0.606 | 1122 | -1 | 1.00 |
| Females: UT | | |  |  |  |  |  |
| Group | Number | Median Days | Median % Increase | Log-rank  P-value | 90th %ile  Days | 90th %ile Increase | Wang-Allison P-value |
| Control | 84 | 906 |  |  | 1100 |  |  |
| FO(50000) | 44 | 892 | -2 | 0.097 | 1067 | -3 | 0.54 |
| FO(15000) | 44 | 831 | -8 | 0.486 | 1109 | 1 | 0.76 |
| NDGA(5000) | 40 | 815 | -10 | 0.053 | 1004 | -9 | 0.22 |

Supplemental Table 3: C2012 Mice, site-specific results for ACA started at 16 months

| Males: TJL | | |  |  |  |  |  |
| --- | --- | --- | --- | --- | --- | --- | --- |
| Group | Number | Median  Days | Median % Increase | Log-rank  P-value | 90th %ile  Days | 90th %ile Increase | Wang-Allison P-value |
| Control | 101 | 844 |  |  | 1006 |  |  |
| ACA | 53 | 876 | 4 | 0.003 | 1170 | 16 | 0.01 |
| Males: UM | | |  |  |  |  |  |
| Group | Number | Median  Days | Median % Increase | Log-rank  P-value | 90th %ile  Days | 90th %ile Increase | Wang-Allison P-value |
| Control | 84 | 870 |  |  | 1111 |  |  |
| ACA | 44 | 937 | 8 | 0.009 | 1191 | 7 | 0.02 |
| Males: UT | | |  |  |  |  |  |
| Group | Number | Median  Days | Median % Increase | Log-rank  P-value | 90th %ile  Days | 90th %ile Increase | Wang-Allison P-value |
| Control | 98 | 751 |  |  | 994 |  |  |
| ACA | 50 | 792 | 5 | 0.23 | 1133 | 14 | 0.09 |

| Females: TJL | | |  |  |  |  |  |
| --- | --- | --- | --- | --- | --- | --- | --- |
| Group | Number | Median  Days | Median % Increase | Log-rank  P-value | 90th %ile  Days | 90th %ile Increase | Wang-Allison P-value |
| Control | 96 | 922 |  |  | 1092 |  |  |
| ACA | 48 | 875 | -5 | 0.56 | 1240 | 14 | 0.25 |
| Females: UM | | |  |  |  |  |  |
| Group | Number | Median  Days | Median % Increase | Log-rank  P-value | 90th %ile  Days | 90th %ile Increase | Wang-Allison P-value |
| Control | 90 | 842 |  |  | 1084 |  |  |
| ACA | 44 | 904 | 7 | 0.23 | 1133 | 5 | 0.23 |
| Females: UT | | |  |  |  |  |  |
| Group | Number | Median  Days | Median % Increase | Log-rank  P-value | 90th %ile  Days | 90th %ile Increase | Wang-Allison P-value |
| Control | 92 | 873 |  |  | 1116 |  |  |
| ACA | 43 | 919 | 5 | 0.19 | 1160 | 4 | 0.11 |

Supplemental Table 4: Comparison of Met/Rapa to Historical Data for Rapa alone

| **Male** |  |  | **Female** |  |  |
| --- | --- | --- | --- | --- | --- |
| **Rx** | **Median**  **(Days)** | **% Change** | **Rx** | **Median**  **(Days)** | **% Change** |
| Control[C2011] | 785 |  | Control[C2011] | 875 |  |
| Met | 847 | 8 | Metformin | 875 | 0 |
| Met/Rapa | 971 | 23 | Met/Rapa | 1079 | 23 |
|  |  |  |  |  |  |
| Rapa [C2006] | 887 | 10 | Rapa [C2006] | 1030 | 18 |
| Rapa [C2009] | 909 | 13 | Rapa [C2009] | 1086 | 21 |
